# A resource of RNA-binding protein motifs across eukaryotes reveals evolutionary dynamics and gene-regulatory function

**DOI:** 10.1038/s41587-025-02733-6

**Published:** 2025-07-25

**Authors:** Alexander Sasse, Debashish Ray, Kaitlin U. Laverty, Cyrus L. Tam, Mihai Albu, Hong Zheng, Yevgen Levdansky, Olga Lyudovyk, Taykhoom Dalal, Kate Nie, Cedrik Magis, Cedric Notredame, Eugene Valkov, Matthew T. Weirauch, Timothy R. Hughes, Quaid Morris

**Affiliations:** 1 Department of Molecular Genetics, University of Toronto, Toronto, Ontario Canada; 2 Donnelly Centre, University of Toronto, Toronto, Ontario Canada; 3 Paul G. Allen School of Computer Science and Engineering, University of Washington, Seattle, WA USA; 4 Vector Institute, Toronto, Ontario Canada; 5 Sloan Kettering Institute, Memorial Sloan Kettering Cancer Center, New York, NY USA; 6 Graduate Program in Computational Biology and Medicine, Weill-Cornell Graduate School, New York, NY USA; 7 National Cancer Institute, National Institutes of Health, Frederick, MD USA; 8 Centre for Genomic Regulation, The Barcelona Institute of Science and Technology, Barcelona, Spain; 9 Universitat Pompeu Fabra, Barcelona, Spain; 10 Center for Autoimmune Genomics and Etiology, Divisions of Allergy and Immunology, Human Genetics, Biomedical Informatics and Developmental Biology, Cincinnati Children’s Hospital, Cincinnati, OH USA; 11 Department of Pediatrics, University of Cincinnati College of Medicine, Cincinnati, OH USA; 12 Ontario Institute for Cancer Research, Toronto, Ontario Canada

**Keywords:** Gene regulation, Machine learning, Evolutionary biology, Computational models, Eukaryote

## Abstract

RNA-binding proteins (RBPs) are key regulators of gene expression; however, their RNA-binding specificities, that is, motifs, have not been comprehensively determined. Here we introduce Eukaryotic Protein–RNA Interactions (EuPRI), a freely available resource of RNA motifs for 34,746 RBPs from 690 eukaryotes. EuPRI includes in vitro binding data for 504 RBPs, including newly collected RNAcompete data for 174 RBPs, along with thousands of predicted motifs. We predict these motifs with an algorithm, Joint Protein–Ligand Embedding, which can detect distant homology relationships and map specificity-determining peptides. EuPRI quadruples the number of available RBP motifs, expanding the motif repertoire across all major eukaryotic clades and assigning motifs to the majority of human RBPs. We demonstrate the utility of EuPRI for inferring post-transcriptional function and evolutionary relationships by identifying rapid, recent evolution of post-transcriptional regulatory networks in worms and plants, in contrast to the vertebrate RNA motif set, which has remained relatively stable after a large expansion between the metazoan and vertebrate ancestors.

## Abstract (web summary)

RNA-binding motifs in eukaryotic proteins are presented in a comprehensive resource.

## Main

RNA-binding proteins (RBPs) bind to transcripts post- and co-transcriptionally to regulate their splicing, polyadenylation, localization, translation and degradation^1^ by recognizing specific RNA sequences, structural elements or both^2,3^. These binding specificities can be represented by mathematical models called motifs, which can score RNA sequences based on their likelihood of containing RBP binding sites^4^. Computational motif-finding methods^5–12^ fit motifs to thousands of bound RNA sequences from large-scale in vitro binding assays, including RNA Bind-n-Seq^13^, RNAcompete^14^ and HTR-SELEX^15^. These in vitro-derived ‘intrinsic binding preferences’^4^, typically recapitulated in vivo^12,16,17^, are essential for interpreting in vivo binding data^18^, interpreting noncoding variants^19^ and assigning function to RBPs^17^. Some motif models incorporate RNA structure features, but the RNA structure preferences of mRNA-binding proteins are very often well modeled by a simple lack of base pairing (that is, nucleotide accessibility) over a short, linear primary sequence motif^5,12^.

Current knowledge of RBP binding preferences is highly biased toward a small number of well-studied RBPs and organisms. Less than 0.1% of all eukaryotic RBPs have any available RNA-binding data, most of which are from mammals or *Drosophila*^15,17,18^. RNA motifs can also be assigned to thousands of other RBPs by simple homology rules; RBPs with at least 70% amino acid sequence identity (hereafter abbreviated AA SID) across their RNA-binding domains (RBDs) usually have nearly identical RNA sequence specificities^17^. However, most uncharacterized RBPs have less than 70% AA SID to any RBP with an assigned motif.

Among eukaryotic RBP families, the RNA recognition motif (RRM) is, by far, the most prevalent sequence-specific RBD, and the K-homology domain (KH) is also quite prevalent^2^. The extreme malleability of the sequence specificity of these domains presumably underlies their evolutionary success. Indeed, there are currently almost no cases in which the evolutionary origin of the sequence specificity of extant RBPs in these classes can be traced or even rationalized.

In principle, ‘recognition code’ homology models (that is, models that relate the identity of particular residues in an RBD to its RNA sequence specificity^20,21^) could improve the sensitivity of the ‘70% rule’ described above, as they have for some classes of transcription factors^22,23^. To be successful, these models require that the modeled RBD class always uses the same set of residues to determine RNA sequence specificity. Besides the less abundant PUF domain^20,21^, however, most RBD classes lack these conserved interfaces. For example, RRMs contain two highly conserved RNA sequence recognition regions, RNP1 and RNP2, but their sequence specificity often depends on residues outside these regions, including linkers and C- and N-terminal domain sequences that recognize specific nucleotides by hydrogen bond interactions^24^. More broadly, the RNA-binding region (RBR) of an RBP commonly contains multiple adjacent RBDs, and these RBDs can interact with one another to form distinct binding interfaces^25,26^. This diversity of RNA recognition interfaces suggests the absence of shared RNA specificity-determining residues for most RBPs^25–27^, precluding the use of classic recognition code techniques^22,23^.

A potential solution is training an adaptive ‘homology model’, which learns a similarity metric that predicts shared RNA sequence preferences^23^. One approach, exemplified by the affinity regression method^28^, is to compute these similarities based on a ‘peptide profile’, which counts short peptide sequences that can be located anywhere within the RBP’s RBR. Affinity regression assigns weights to each peptide, which are used in computing the similarity between the profiles of two RBRs. Once defined, this similarity measure can be used to infer the RNA sequence preferences of an uncharacterized protein based on the known RNA preferences of similar RBRs. Due to the small number and biased representation of RBPs with known motifs for the adapted similarity metric, only a small fraction of RBPs have been confidently assigned RNA motifs using such methods.

To address these challenges, we generated new binding data for 174 eukaryotic RBPs and an algorithm that learns a homology model based on peptide profiles. This algorithm, Joint Protein–Ligand Embedding (JPLE), uses representation learning^29^ within a self-supervised linear autoencoder framework^30^ to adapt its homology model. Combining the new binding data with existing in vitro RNAcompete data^17^, we used JPLE to reconstruct RNA motifs and predict RNA-contacting residues for RRM- and KH-domain RBPs across 690 eukaryotes, resulting in reconstructed motifs for 28,283 RBPs with previously uncharacterized RNA-binding specificities. Combining these predicted motifs with other published datasets and additional inferred motifs, we introduce a resource of 34,746 motifs called Eukaryotic Protein–RNA Interactions (EuPRI), made available via our updated CisBP-RNA web tool at https://cisbp.org/rna. Using EuPRI and JPLE, we examined the evolution of extant RBP motifs by identifying groups of RRM- and KH-domain-containing RBPs with a shared, conserved motif and found that most RNA motifs appeared recently in multicellular organisms, with clade-specific gain rates, including rapid expansion of motif vocabularies in Nematoda and Angiospermae. Finally, to demonstrate the utility of this resource, we used JPLE-assigned motifs to identify a group of homologous CID RBPs that regulate mRNA stability in *Arabidopsis thaliana* and perform a deadenylation assay to validate the predicted role of CID8.

## Results

### New RNA motifs for 174 phylogenetically diverse RBPs

**Fig. 1:**
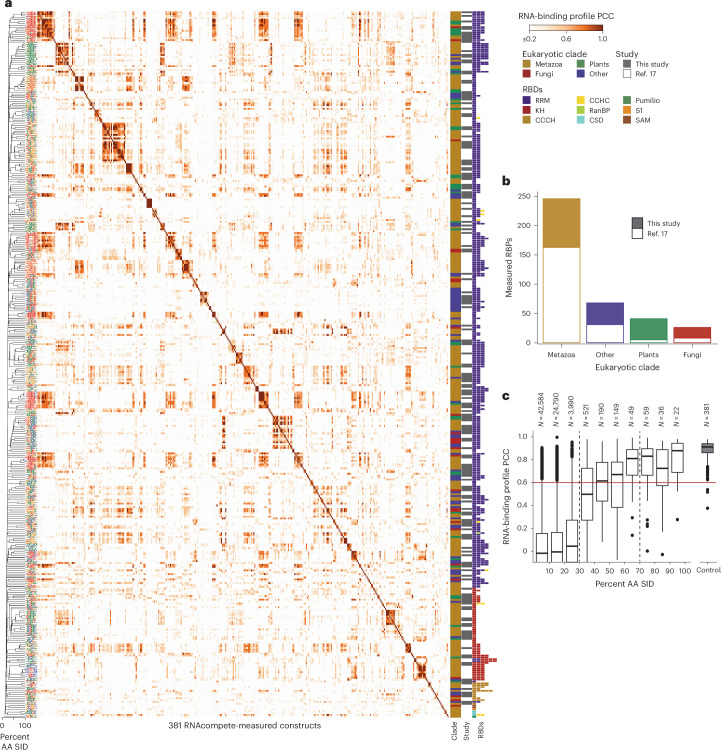
RNAcompete-measured RNA sequence specificities. **a**, Symmetric heat map displaying PCCs of RNAcompete RNA-binding profiles for each pair of RNAcompete-measured constructs. RBPs are clustered by the AA SID of their RBRs using single-linkage hierarchical clustering. Left: logos of position frequency matrices derived from the top ten RNAcompete 7-mers. Right: eukaryotic clade for each RBP, whether it is newly measured in this study, and the count and class of RBDs in the construct. **b**, Count of RNAcompete-measured RBPs across major eukaryotic clades for RBPs measured for this study and measured previously. The ‘other’ category encompasses all species outside of metazoa, fungi and land plants, including algae, excavates, amoebozoa, and Stramenopiles, Alveolates and Rhizarians supergroup species. **c**, Distribution of RNA-binding profile PCCs for pairs of RNAcompete-measured RBPs whose RBRs fall within the AA SID range indicated on the *x* axis. As a control, the distribution of PCCs between RNAcompete Set A and Set B for the same experiment are displayed to the right. Pairs of RBPs with a PCC of >0.6 (above the red line) are considered to have similar RNA specificities, whereas pairs of RBPs with a PCC of <0.6 have dissimilar RNA specificities. The number of RBP pairs (*N*) in each AA SID range is indicated above each box. Boxes span the interquartile range (IQR), with the center line marking the median. Whiskers span from minimum to maximum value within IQR × 3 / 2 from box boundaries. Outliers are displayed as dots.

**Extended Data Fig. 1:**
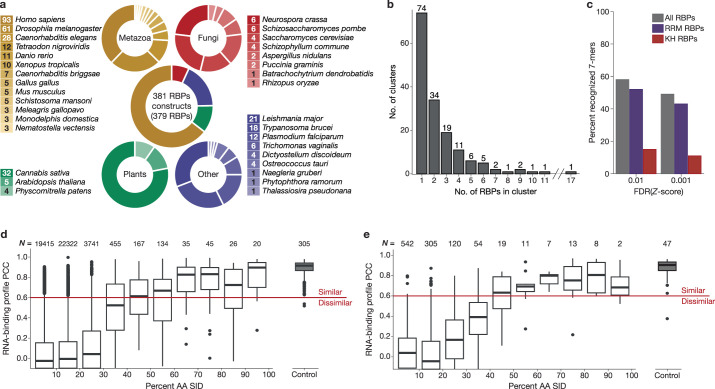
RNAcompete- measured RNA sequence specificities. **a**, The center donut chart depicts the breakdown of RNAcompete-measured RBPs across major eukaryotic clades and 33 species, including both the RBPs measured for this study and those from Ray et al.^17^. Surrounding donut charts depict the breakdown of RBPs by species for metazoa, fungi, plants, and other clades. The “other” category encompasses all species outside of metazoa, fungi, and land plants, including algae, excavates, amoebozoa, and SAR (Stramenopiles, Alveolates, and Rhizarians) supergroup species. Legends adjacent to the donut charts show the number of measured RBPs for individual species. **b**, The RNAcompete-measured RBPs were split into clusters based on RNA-binding profile similarity; sequence specificities were hierarchically, agglomeratively clustered on the Pearson correlation coefficients (PCCs) between RNA-binding profiles with complete linkage. Using a PCC cut-off of 0.6, 157 clusters were identified (Table S1) and the distribution of their sizes is displayed. **c**, Percentage of all 7-mers that are “specifically bound”, or recognized, (one-sided Z-test), FDR <0.01 or < 0.001 (Benjamini-Hochberg FDR correction over the 16382 7-mers), by at least one RNAcompete-measured RBP, or at least one RNAcompete-measured RRM- or KH-domain RBP. **d**, **e**, Box plots show the distribution of RNA-binding profile PCCs for pairs of RBPs whose RBRs fall within the percent amino acid sequence ID (AA SID) range indicated on the x- axis. **d** depicts only the 308 RRM-domain-containing RBPs and **e** depicts only the 47 KH-domain-containing RBPs. As a control, the distribution of PCCs between RNAcompete Set A and Set B for the same experiment are displayed to the right. The number of RBP pairs (*N*) in each AA SID range is indicated above each box. Boxes span the interquartile range (IQR) with the center line marking the median. Whiskers span from minimum to maximum value within IQR*3/2 from box boundaries. Outliers are displayed as dots.

To derive accurate RNA motifs for as many eukaryotic RBPs as possible, we generated new RNA-binding data for RBPs selected to provide useful training data for our homology models while also maximizing the number of RBPs to which we could confidently assign motifs. We selected proteins from 45 well-annotated eukaryotes^31^ using a semiautomated procedure to identify potential RBPs containing one or more conventional RBDs that (1) have many other proteins within 70% AA SID of their putative RBR, that is, the protein subsequence containing all of the predicted RBDs, (2) represent both model organisms and under-represented eukaryotic clades and (3) would, when combined with pre-existing RNAcompete data^17^, provide a more uniform coverage of pairwise protein AA SID levels between measured RBPs. This process produced an initial set of 277 proteins. We measured the intrinsic binding preferences of these candidate RBPs using RNAcompete^17^ and identified a subset of 174 RBPs with high-quality data using a rigorous, semiautomated quality control procedure^32^, thus establishing sequence-specific RNA-binding function for these RBPs. In combination with pre-existing RNAcompete data for an additional 205 RBPs^17^, we established a resource of experimentally measured RNA-binding specificities for 379 RBPs (across 381 constructs). This dataset provides broad coverage of RBD architectures and major eukaryotic clades, including 41 plant RBPs (Fig. 1a,b, Extended Data Fig. 1a and Supplementary Table 1).

Standard quantification of RNAcompete data produces an estimate of the relative binding affinity (also known as the ‘*z* score’) of an RBP to every possible RNA 7-mer (ref. ^32^). We refer to the vector containing these 4^7^ (=16,384) *z* scores as the RBP’s RNA-binding profile and use it as a detailed representation of the RNA motif implied by the RNAcompete data. We use the Pearson correlation coefficient (PCC) of RNA-binding profiles to measure the similarity of two RNA motifs (Fig. 1a).

Two lines of evidence support the success of our experimental strategy. First, our strategy for choosing diverse RBPs produced a broad range of RNA sequence specificities; the dataset contains 157 distinct clusters of RNA motifs (Supplementary Table 1). In contrast to a previous report^16^, the data contain a wide diversity of RNA motifs, particularly among the RRM-containing proteins; nearly half of the clusters (*n* = 74) contain only one RBP (Extended Data Fig. 1b), and among the 306 RRM-containing proteins, up to 53% of all possible 7-mers are specifically bound by at least one RBP (Benjamini–Hochberg-adjusted *z*-score *q* value of <0.01; Extended Data Fig. 1c). Second, the previously reported separation of pairs of RBPs into 3 classes (highly similar (>70% AA SID, PCC > 0.6), variable (30–70% AA SID, variable PCC) and dissimilar (<30% AA SID, PCC < 0.6) pairs) was recapitulated (Fig. 1c), even when considering RRM and KH domains individually (Extended Data Fig. 1d,e). Thus, this resource provides a broad coverage of the space of possible RNA targets while demonstrating that the previously reported relationship^17^ between protein AA SID and RNA motif similarity holds across eukaryotes.

The RNAcompete data illustrate the challenge of inferring RNA sequence specificity by amino acid sequence homology alone, particularly among the large group of RBP pairs with 30 to 70% AA SID. In this range, AA SID ceases to be a reliable measure of motif similarity; the RBP pairs in this range are nearly equally divided among those with high and low motif similarity (Fig. 1c).

### JPLE algorithm

**Fig. 2:**
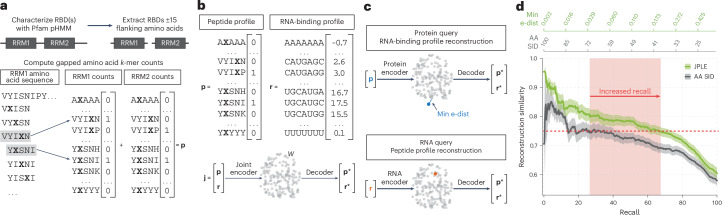
JPLE captures the association between amino acid sequence and RNA sequence specificity. **a**, Derivation of the peptide profile of an RBP. The amino acid sequences of each RBD within the RBP are extracted, along with 15 flanking amino acids, and the occurrence of each amino acid 5-mer with a single wildcard character (*X*) in position 2, 3 or 4 is counted. An RBP’s peptide profile is the vector of gapped peptide 5-mer counts summed across all its RBDs. **b**, The peptide profile (**p**) is concatenated with the RNA-binding profile (**r**) of an RBP to produce a joint vector (**j**). The joint encoder maps from **j** to a low-dimensional embedding, *W*, and a decoder function maps from *W* to reconstructions p* and r* of the peptide profile and RNA-binding profile, respectively. **c**, The encoder function can be used with partial input: protein queries (top) estimate *W* from **p** using a protein-only encoder, and RNA queries (bottom) estimate *W* from **r** using an RNA-only encoder. Cosine distance in the embedding space to the closest RNAcompete-measured RBP embedding (minimum e-dist) is used to assign confidence to this reconstruction. Min, minimum. **d**, Precision–recall curves for RNA-binding profile reconstructions generated by AA SID and JPLE. Standard error is shown in the shaded area around each line. Precision (*y* axis) is the mean PCC for reconstructions at least as confident as the threshold (top axes). AA SID confidence is AA SID, and JPLE confidence is the minimum e-dist. The left boundary of the highlighted region indicates the recall at an AA SID threshold of 70%, at which a mean PCC of 0.75 is achieved. The right boundary of the highlighted region indicates the recall of JPLE at a PCC of 0.75.

Taking advantage of the expanded repertoire of motifs, we sought to improve on the prediction of RNA motifs for uncharacterized RBPs at a greater evolutionary distance, that is, lower sequence homology. To do this we developed JPLE, a homology model based on peptide profiles. JPLE captures the association between amino acid sequence and RNA sequence specificity by learning a mapping between (1) a vector, **p** (that is, the peptide profile of the RBP), representing the count of each short peptide observed in the RBR of the RBP and (2) a vector, **r** (that is, the RNA-binding profile), representing the RNA motif as a table of scores for all possible *k*-mers. In the current implementation of JPLE, **p** consists of entries for all possible 5-mer peptides, with one wildcard character, present within the RBRs of the RNAcompete-measured proteins (Fig. 2a), whereas ***r*** consists of the 7-mer *z* scores derived from RNAcompete.

**Extended Data Fig. 2:**
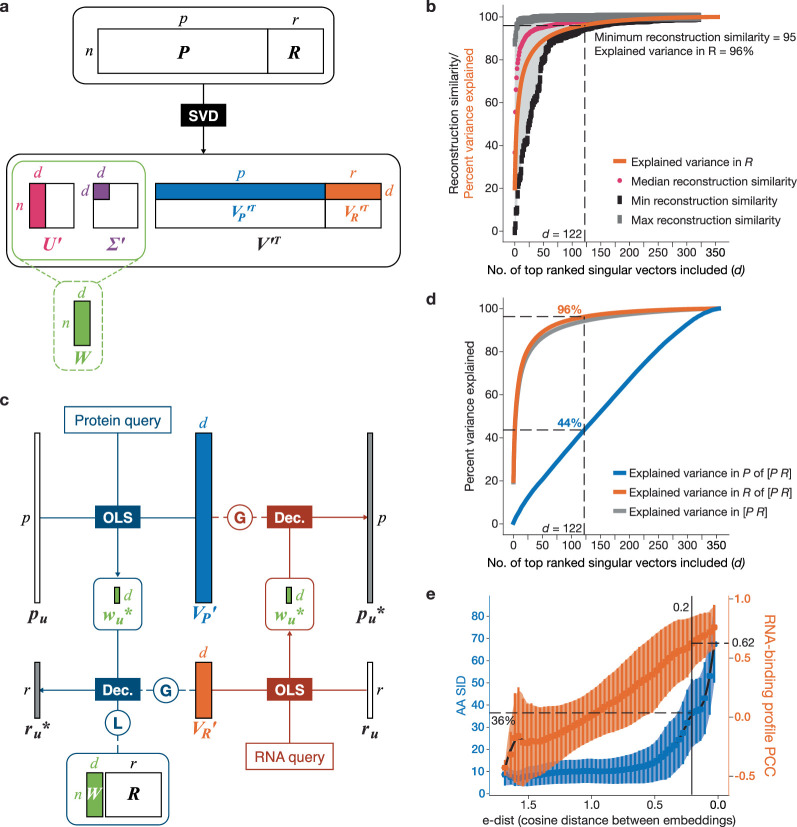
JPLE captures the association between amino acid sequence and RNA sequence specificity. **a**, Illustration of the JPLE training process for *n* RBPs. Singular value decomposition (SVD) is used to decompose the joint protein representation **[*P R*]** into ***U***, ***Σ***, and ***V***^***T***^. The *d* singular vectors and values contributing the most to the variance of ***R*** in **[*P R*]** are selected, leading to the submatrices ***U’***, ***Σ’***, and ***V’***^***T***^. The product ***W*** of ***U’*** and ***Σ’*** provides the *d*- dimensional latent embedding of the *n* RBPs. **b**, Distribution of the Pearson correlation coefficients (PCCs) between the reconstructed (r*) and measured (***r***) RNA-binding profiles (that is, the reconstruction similarity), as a function of the number of maintained singular vectors *d*. Note that PCC is multiplied by 100. The median, minimal, and maximal reconstruction similarities are displayed, and the distribution is indicated in gray. To enable a minimum reconstruction PCC of 0.95 for all measured RBPs, *d* = 122 is required. The orange line represents the percentage of variance explained in ***R*** of **[*P R*]**. **c**, Illustration of the JPLE inference process for RBP_*u*_. The left (in blue) showcases a protein query, where the RBP’s latent embedding ***w***_u_* is obtained by deconvolving its peptide profile ***p***_***u***_ into a mixture of the singular vectors in ***V***_***P***_***’***. Its RNA-binding profile ***r***_u_* can be reconstructed through either global (labeled G) or local (labeled L) decoding. The right (in brown) showcases RNA query, where the RBP’s latent embedding ***w***_u_* is obtained by deconvolving its RNA-binding profile ***r***_***u***_ into a mixture of the singular vectors in ***V***_***R***_***’***. Its peptide profile ***p***_u_* can be reconstructed through global decoding. **d**, Variance explained in ***P*** and ***R*** of **[*P R*]**, as a function of the number of selected singular vectors *d*. At *d* = 122, 44% and 96% of the total variance of ***P*** and ***R*** of **[*P R*]** are explained respectively. **e**, Relationship between RNA-binding profile PCCs and their JPLE latent distance. JPLE was trained leaving clusters of RBPs with the same specificity (PCC > 0.6) out, then embedding them into JPLE and measuring the cosine distances between each other and to the RBPs in the training set (that is, the e-dist). The e-dist for each pair of RBPs was compared to the similarity of their RNA-binding profiles (orange, right y-axis) and their amino acid sequence identity (blue, left y-axis). Lines and shaded areas show smoothed mean and standard deviation across 50 equally sized bins. RBP pairs with an e-dist < 0.20 possess an average RNA-binding similarity of at ≥ 0.62 and an average AA SID ≥ 36%.

In JPLE, the mapping between the peptide profile of a protein, **p**, and its RNA-binding profile, **r**, is made using a low-dimensional embedding, *W*. The embeddings are computed from joint vectors, which contain both **p** and **r**, via a joint encoder that is trained using a modification of principal component analysis (PCA; Fig. 2b and Extended Data Fig. 2a). In standard PCA, a dataset of high-dimensional vectors is transformed into a new, orthogonal, low-dimensional coordinate system (that is, an embedding) that captures most of the variation in the initial dataset. In JPLE, the high-dimensional vectors are the joint vectors derived from the RNAcompete-measured RBPs. In PCA, the new coordinate system is represented by an orthonormal set of high-dimensional vectors, called principal axes, and the coordinates of the low-dimensional embedding of a vector is computed by calculating its projection on each of the principal axes. JPLE’s joint encoder is identical to PCA, except that JPLE’s principal axes are selected only based on their ability to capture the variation in the RNA-binding profile rather than the joint vector.

Trained on the 355 joint vectors representing each of the 355 RNAcompete-measured RBP constructs that contain only RRM and/or KH domains, JPLE required only 122 axes to explain 96% of the variance in *R* (the matrix of all 355 **r** vectors), that is, its low-dimensional embedding space has 122 dimensions (Methods and Extended Data Fig. 2d). This is a substantial reduction in dimensionality compared to that of each joint vector (*n* = 131,889) and even the number of joint vectors (*n* = 355). By considering only RNA-binding profiles when selecting principal axes, JPLE’s encoder is effectively suppressing the impact, on the embedding, of peptides that have no association with RNA-binding specificity. Overall, JPLE’s embedding only captures 44% of the variance in the peptide profiles (Extended Data Fig. 2d), and, as we illustrate below, the captured peptide variations tend to correspond to RNA-contacting peptides that determine RNA specificity.

Once the joint encoder is defined, we can analytically define encoders that compute optimal least squares estimates of the corresponding embedding from any predefined subset of the features in the joint vector, for example, all the peptide features (Methods). JPLE defines both a protein encoder that estimates embeddings using only the protein-encoding features in the joint vector (that is, **p**) and an RNA encoder that uses only the RNA motif-encoding features (that is, **r**; Fig. 2c and Extended Data Fig. 2c). An embedding computed from any of the encoders can be decoded, reconstructing the high-dimensional joint vector from a weighted combination of the JPLE principal axes, where the weights are derived from the embedding (Fig. 2b).

We found that the similarity between a protein encoder-computed embedding of a held-out RBP and a training set RBP embedding is an accurate predictor of the correlation of their RNA-binding profiles (Extended Data Fig. 2e). We define the embedding distance (e-dist) between two RBPs as 1 minus the cosine similarity of their embeddings, and we use low e-dist as a replacement for high AA SID to determine whether two RBPs have the same RNA motif. The e-dist can be used to assign RNA motifs to uncharacterized RBPs via protein query (Fig. 2c and Extended Data Fig. 2c). This query uses the protein encoder to embed an uncharacterized RBP’s peptide profile, uses e-dist to identify its nearest neighbors among the embeddings for the RNAcompete-measured RBPs and estimates the RNA-binding profile of the queried RBP as a weighted average of RNA-binding profiles of these nearest neighbors. JPLE therefore provides many of the advantages of traditional PCA: it provides a minimal representation of a dataset, the embeddings themselves are meaningful, and it suppresses noninformative variation (that is, peptides that do not confer specificity). In subsequent sections, we use JPLE, and the RNA-binding profile it reconstructs, to address a series of problems in the function and evolution of RBPs and RBP motifs.

### JPLE doubles the number of RBPs assigned RNA motifs

We used a leave-one-out cross-validation framework to assess the reconstruction accuracy of JPLE’s protein queries. Figure 2d shows average reconstruction accuracy as a function of increasing e-dist to the closest RBP embedding. Also shown is the average accuracy as a function of increasing AA SID for a simple homology model where the predicted RNA-binding profile is that of the training set RBP with the highest AA SID (that is, its AA SID nearest neighbor). Thresholding the simple homology model at 70% AA SID recapitulates our previous approach for inferring motifs^17^. This cutoff has an average RNA-binding profile reconstruction accuracy of PCC = 0.745 and a recall of 27.6%. At an e-dist cutoff of 0.2, JPLE has similar average PCC (=0.748) and a 67.6% recall, a 2.4-fold increase. This gain in recall is equivalent to being able to reconstruct motifs for all held-out RBPs with at least 40% AA SID to an RBP in the embedding (Fig. 2d and Supplementary Table 2).

**Extended Data Fig. 3:**
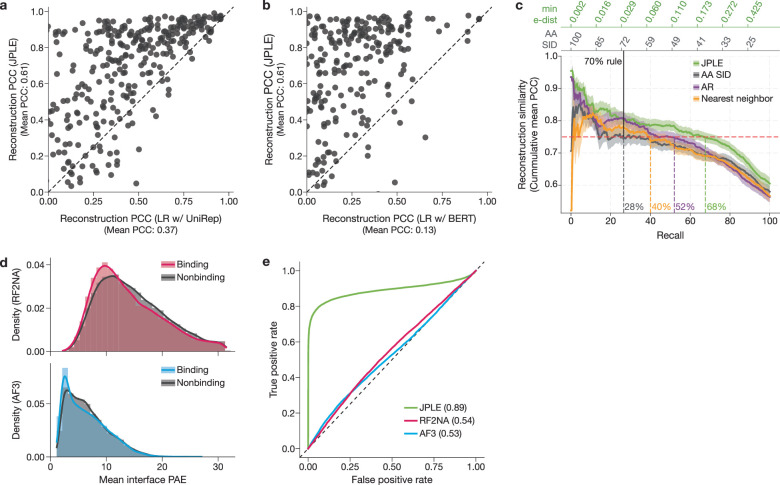
JPLE outperforms alternative methods. **a**, **b**, Comparison of the RNA-binding profile reconstructions generated by JPLE trained with 5-mer peptide features, to those generated by two linear regression (LR) models trained with deep learning features from TAPE: UniRep (**a**) and BERT (i.e., Transformer) (**b**). As in Extended Data Fig. 2, reconstruction PCCs are computed between the reconstructed (r*) and measured (***r***) RNA-binding profiles. **c**, Precision-recall curves for RNA- binding profile reconstructions generated by JPLE, AA SID, affinity regression (AR), and the nearest neighbor model (see **Methods**). Precision (y-axis) shows the mean PCC for reconstructions at least as confident as the threshold (top axes). JPLE confidence is e-dist to the nearest neighbor; AA SID confidence is % amino acid identity; AR confidence is one minus PCC between the test and nearest neighbor’s embedding; the nearest neighbor model confidence is e-dist to the nearest neighbor. At the AA SID threshold of 70%, a mean PCC of 0.75 is achieved (red line). The recall for all four methods at a mean PCC of 0.75 is indicated. Standard error is shown in the shaded area around each line. **d**, Distribution of the mean interface predicted aligned errors (PAEs) for RoseTTAFold2NA (RF2NA) and AlphaFold3 (AF3) predicted structures with high-affinity 7-mers (binding) and low-affinity 7-mers (nonbinding) for all 355 RBPs. **e**, ROC curves compare the performance of RF2NA, AF3, and JPLE in the task of differentiating high-affinity from low- affinity 7-mers. The predictions for RF2NA and AF3 are the mean interface PAEs (see **d**). The predictions for JPLE are the predicted *z* scores on held-out RBPs. Numbers contained within brackets in the legend display the AUROCs.

To illustrate the value of JPLE’s embedding technique, we compared the performance of JPLE to alternative methods with differing protein sequence representations or differing methodology for computing the embedding. First, we evaluated replacing JPLE with linear models trained on fixed-length protein representations derived using the application of modern natural language processing methods to large protein sequence databases^33^. These fixed-length profiles led to substantially lower PCCs between real and reconstructed RNA-binding profiles (Extended Data Fig. 3a,b and Supplementary Table 2). Next, we evaluated the effect of replacing JPLE’s embeddings with the PCA-based embeddings used by affinity regression^28^; this approach had substantially lower recall at the average PCC = 0.75 cutoff (Extended Data Fig. 3c and Supplementary Table 2). We also evaluated RoseTTAFold2NA (RF2NA)^34^ and AlphaFold 3 (AF3; ref. ^35^) but found that their ability to differentiate between high- and low-affinity 7-mers was barely distinguishable from random (Extended Data Fig. 3d,e). These comparisons underline the importance of JPLE’s strategy to represent protein sequences using peptide profiles and to learn protein sequence embeddings using joint vectors that include RNA specificity information.

### JPLE predicts RNA specificity-determining residues

The success of JPLE protein queries in predicting RNA motifs suggests that the embeddings for uncharacterized RBPs are encoding the protein sequence features important for target recognition. To investigate what these features are, and how they support RNA motif predictions, we used JPLE RNA queries to reconstruct peptide profiles for known RNA-binding profiles and identified the residues that are represented in the embedding.

Using the RNA encoder, we transformed RNA-binding profiles into the embedding space and reconstructed the peptide profiles (Fig. 2c and Extended Data Fig. 2c). Because the joint encoder suppresses information about peptides not associated with the RNA-binding profile, and the peptide profiles used to train the joint encoder were counts, we expect the reconstructed peptide profile to contain high values for peptides informative about the RNA specificity of the associated RBP. In practice, because peptides in the profile contain wildcard characters (see ‘JPLE’; Fig. 2a), identifying informative peptides is more complicated, but we can still obtain a residue importance score^28^ (RIS) for each residue by summing scores from each peptide it appears in. The RIS thus measures the degree to which the identity of a given residue is encoded in the embedding associated with its RNA specificity.

**Fig. 3:**
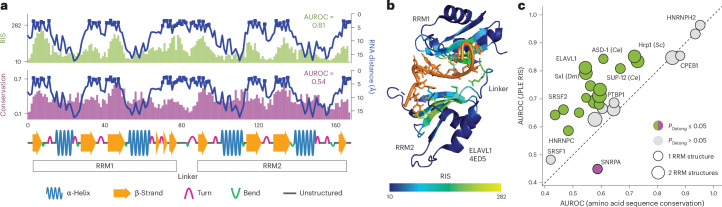
JPLE predicts RNA-interacting amino acids. **a**, The distance between individual amino acid residues and RNA (in angstroms) is compared to JPLE RIS (top) and conservation scores (middle) for the RBP–RNA cocomplex structure in **b**. RNA-contacting residues (that is, within 5 Å of the RNA) are indicated by dots. A linear visualization of the protein secondary structure is depicted at the bottom. **b**, The RBP–RNA cocomplex structure (PDB 4ED5) depicts the two N-terminal RRMs from ELAVL1 (*Homo sapiens*), with regions colored by JPLE RIS. **c**, Comparison between sequence conservation and JPLE RIS for predicting RRM domain interface residues (that is, the distance from RNA), evaluated with AUROC. Colored circles indicate a significant difference (*P* < 0.05) in the AUROC between the two scoring methods, as determined by the Delong test (two-sided). *Ce*, *C. elegans*; *Dm*, *D. melanogaster*; *Sc*, *Saccharomyces cerevisiae*.

**Extended Data Fig. 4:**
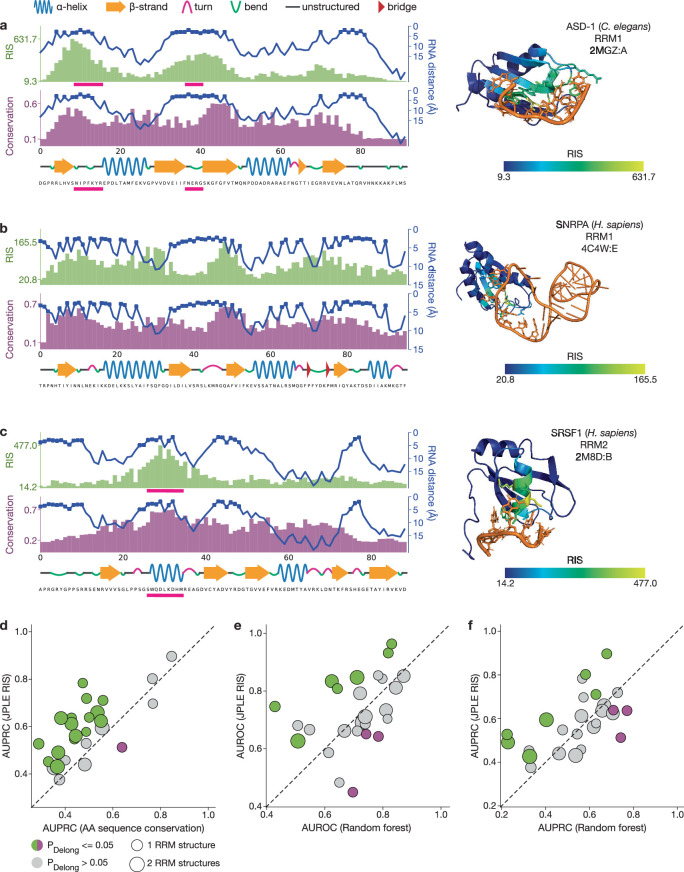
JPLE predicts RNA- interacting amino acids. **a-c**, The distance between individual residues and RNA (in angstroms) is compared to JPLE residue importance scores (RISs) (top left) and conservation scores (middle left) for the RRM-RNA cocomplex structure depicted to the right. RNA-contacting residues (that is, within 5 Å of the RNA) are indicated by dots in the plots on the left. A linear visualization of the protein secondary structure is depicted at the bottom left along with the protein sequence. RRM-RNA cocomplex structures are coloured by JPLE RIS. The loops between β1 and α1 and between β2 and β3 found to confer specificity in RBFOX1^38^, the human homolog of *C. elegans* ASD-1, are indicated in **a** with a pink bar below the amino acid sequence and below the RIS histogram. Similarly indicated in **c** is the α-helix that confers sequence specificity in the depicted SRSF1 RRM^39^. **d**, Comparison between sequence conservation and JPLE RISs for predicting RRM domain interface residues, evaluated with AUPRC. Coloured circles indicate a significant difference (P < 0.05) in AUROC between the two scoring methods, as determined by the Delong test (two-sided) using corresponding ROC curves (see Fig. 3c). **e-f**, Comparison between JPLE RIS and a random forest model trained using the following features: amino acid position specific sequence matrices from a multiple sequence alignment, physico-chemical residue features, residue identity, and conservation within a window of five amino acids. Results of evaluation by AUROC are shown in **e**, and by AUPRC in **f**. Points in both panels are coloured according to significance of the Delong test (two-sided) performed on the ROC curves.

We derived RISs for 26 RNAcompete-measured RRM-containing RBPs that had an RRM–RNA cocomplex structure in the Protein Data Bank (PDB^36^; Methods), mapped the RIS values onto the structures and visualized the result (for example, Extended Data Fig. 4a–c). In general, RIS values were highest for RNA-contacting residues, and, often, they were highest for residues that interact with RNA bases rather than the RNA backbone. For example, in human ELAVL1 (also known as HuR), high RIS values were observed for RRM1 and RRM2 β-sheets and the linker region between the RRMs, consistent with structural analyses of ELAVL1/HuR^37^ (Fig. 3a,b). For *Caenorhabditis elegans* ASD-1, an ortholog of the human RBFOX proteins, JPLE assigns higher scores than conservation to residues within two loops that establish contacts to the first four nucleotides of its preferred UGCAUG motif^38^ (Extended Data Fig. 4a). Human proteins SNRPA and SRSF1 provide two examples for which RIS values were highest only for those RNA-contacting residues that determine primary RNA sequence specificity. SNRPA, a stem-loop binding protein, was assigned high RIS values for residues contacting single-stranded RNA in the loop and low RIS values for conserved residues that contact the double-stranded RNA stem (Extended Data Fig. 4b). For SRSF1, JPLE assigns high RIS values to the specificity-determining contacts in one of the pseudo-RRM’s α-helices^39^ and low RIS values to N-terminal residues that contact the RNA backbone (Extended Data Fig. 4c).

To quantitatively assess these initial observations that JPLE’s RNA queries identify RNA interfaces, we evaluated the ability of RIS values to classify RNA-contacting residues (that is, those within 5 Å of the RNA), using the area under the receiver operating characteristic (ROC) curve (AUROC) and compared their performance to 2 baselines: sequence conservation and a random forest model trained on the 26 RRM–RNA cocomplex crystal structures (Methods). JPLE’s RIS values were better than conservation at identifying RNA-contacting residues for almost all 26 RBPs and significantly better for 16 (*P* < 0.05, DeLong test; Fig. 3c, Extended Data Fig. 4d and Supplementary Table 3). RIS values were significantly worse only for SNRPA, where many of the RNA-contacting residues recognize RNA structure, which is not encoded in the RNA-binding profiles. RIS values had, on average, higher AUROCs than the random forest classifier, with significantly better AUROCs for seven RBPs and significantly worse AUROCs for three, including SNRPA (Extended Data Fig. 4e,f). Collectively, these observations suggest that JPLE’s embeddings are encoding the identity of RNA specificity-determining residues for diverse, and RBP-specific, sets of RBP–RNA interfaces and that JPLE can provide useful structural information for RBPs without known RNA interfaces.

### The EuPRI resource contains motifs for 34,746 RBPs

**Fig. 4:**
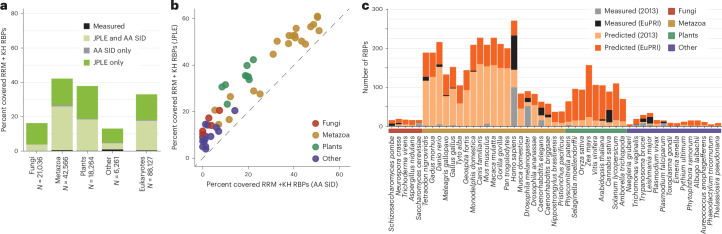
JPLE reconstructs RNA-binding specificities for thousands of eukaryotic RBPs. **a**, The percentage of measured and predicted specificities for RRM- and KH-domain RBPs across 690 species is shown across major eukaryotic clades and for all eukaryotes combined. The proportions of predicted specificities that were identified by AA SID, JPLE or both are indicated. **b**, Scatter plot displaying the percentage of specificities for RRM- and KH-domain RBPs that were reconstructed by JPLE compared to AA SID with the 70% rule for 49 representative species (listed in **c**). **c**, The number of measured and predicted (that is, reconstructed or inferred) RBP specificities in EuPRI for 49 eukaryotes. Newly measured and predicted specificities are differentiated from the measured and predicted motifs available in the 2013 release of the CisBP-RNA database^17^.

**Extended Data Fig. 5:**
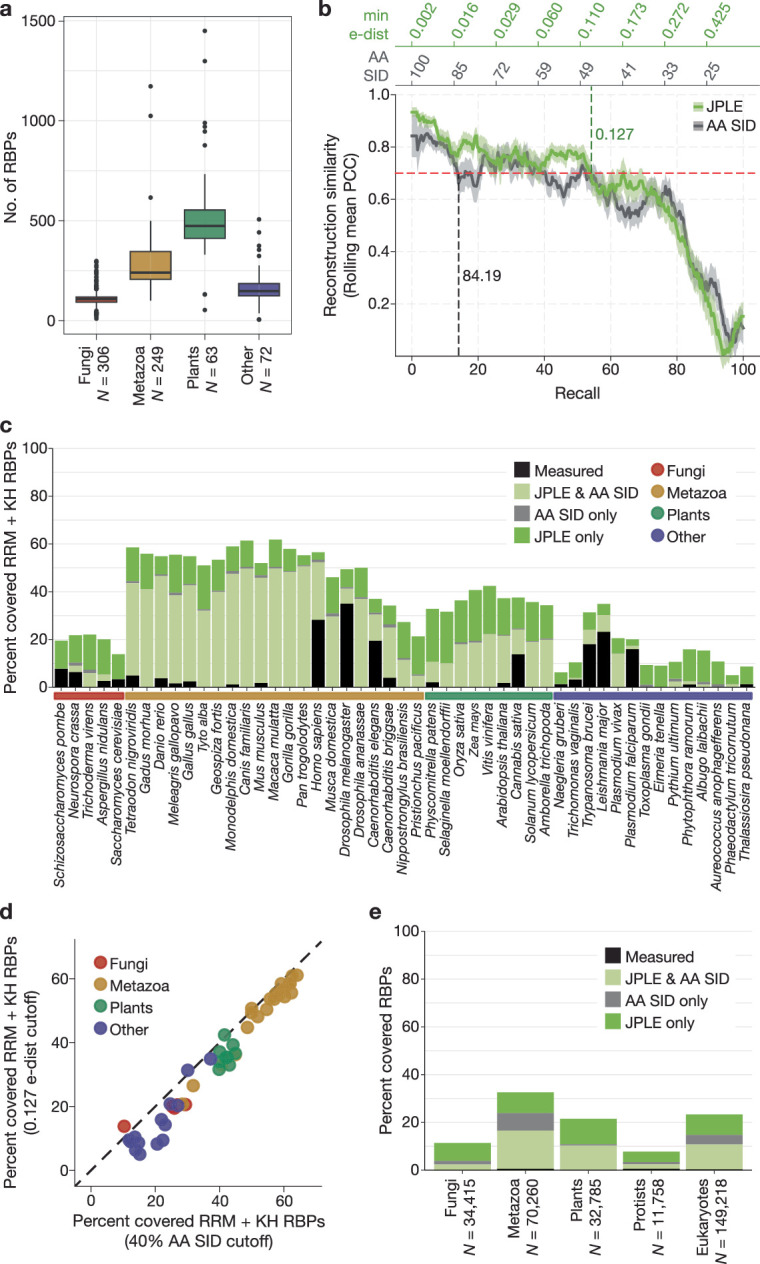
JPLE reconstructs RNA-binding specificities for thousands of eukaryotic RBPs. **a**, The number of RBPs identified in each of 690 eukaryotes split across major eukaryotic clades. Boxes span the interquartile range (IQR) with the center line marking the median. Whiskers span from minimum to maximum value within IQR*3/2 from box boundaries. Outliers are displayed as dots. **b**, Precision-recall curves for RNA-binding profile reconstructions generated by amino acid sequence identity (AA SID) and JPLE. Precision (y-axis) is the mean rolling Pearson correlation coefficient (PCC) for reconstructions at least as confident as the threshold (top axes). The selection size for the rolling average window is 25 reconstructions. AA SID confidence is percent amino acid identity, JPLE confidence is the minimum e-dist. Grey and green dashed lines indicate the confidence at which the rolling mean PCC first hits 0.70 (red dashed line). Standard error is shown in the shaded area around each line. **c**, The fraction of measured and reconstructed specificities for RRM- and KH-domain-containing RBPs for 49 representative species. The proportion of reconstructed specificities that were identified by AA SID, JPLE, or both are indicated. The clade to which the species belongs is indicated below the x-axis. **d**, Scatterplot displays the percentage of specificities for RRM- and KH-domain-containing RBPs that were reconstructed by JPLE (with an e-dist cutoff of 0.127) compared to a 40% AA SID for 49 representative species (listed in panel **c**). **e**, The fraction of measured and reconstructed RBP specificities for major eukaryotic clades and all eukaryotes contained in EuPRI and on CisBP-RNA. This plot includes measured and reconstructed RBPs that do not contain RRM- or KH-domain-containing RBPs.

Next, we took advantage of the fact that JPLE can provide reconstructions of RNA motifs for RBPs that are not in the training set, achieving the same reconstruction accuracy for twice as many proteins as homology-based inference at previously established thresholds^17,31^ (Fig. 2d). We applied JPLE to assign RNA motifs to as many RBPs as possible among 690 sequenced eukaryotes. For comparison, we also assigned motifs using the 70% rule. We performed protein queries for all KH- and RRM-containing (candidate) RBPs in the predicted proteomes of all 690 genomes (Extended Data Fig. 5a and Supplementary Table 4) and assigned RNA motifs to those whose e-dists to an RNAcompete-measured RBP were below a stringent e-dist cutoff of 0.127. This cutoff guarantees not only an overall average PCC of 0.75 on held-out RBPs but also a rolling average of PCC > 0.70 at all levels of recall up until the cutoff (Extended Data Fig. 5b). Of the 76,903 RRM-only RBPs, 10,917 KH-only RBPs and 307 RBPs that contained both domains, JPLE assigned RNA motifs to 24,320 (32%), 3,749 (34%) and 248 (81%) unmeasured RBPs, respectively (Fig. 4a), with particularly large gains for nonmetazoan eukaryotes (Fig. 4b and Extended Data Fig. 5c). As anticipated by our cross-validation studies, the coverage achieved by JPLE is equivalent to being able to assign RNA sequence specificity to every RRM- and KH-domain-containing RBP with at least 40% AA SID to an RNAcompete-measured RBP (Extended Data Fig. 5d).

To generate a comprehensive resource of eukaryotic RNA-binding specificities, we combined the RNAcompete-measured and JPLE-reconstructed motifs with motifs reported in other large in vitro selection studies^15,16^. Using the 70% rule against all 504 RBPs with measured motifs, we inferred RNA motifs for a further 5,959 RBPs beyond those described above, the majority of which contain RBDs that lack sufficient training data for JPLE (for example, the CCCH zinc finger domain). We deposited this resource, called EuPRI, in our CisBP-RNA database (https://cisbp.org/rna).

Between directly measured motifs, JPLE-reconstructed motifs and motifs inferred with the 70% rule, EuPRI provides sequence specificities for 34,746 eukaryotic RBPs. For the 28,667 RNA sequence specificities reconstructed by JPLE, we further performed JPLE RNA queries, thereby assigning a RIS to each residue in the associated RBR and report these values on CisBP-RNA. Together, EuPRI provides specificities for about 33% of RBPs from metazoa, 21% from plants and 10% from fungi and other eukaryotes (Extended Data Fig. 5e). For humans, EuPRI provides specificities for 196 RBPs with RRM and KH domains, representing 69.5% of all RBPs with these domains. The largest increase in the absolute number of new motifs is in plants; EuPRI adds, on average, measured or predicted motifs for 111 RBPs per plant species and 114 for angiosperms (Fig. 4c and Supplementary Table 4). EuPRI also covers up to 30% of RBPs for important clades of human parasitic organisms, including *Leishmania major* and *Trypanosoma brucei*.

### Age and evolution of RNA motifs

EuPRI’s comprehensive coverage of the eukaryotes, together with JPLE’s accuracy at detecting remote homology, provide a unique opportunity to investigate the evolution of RNA specificity. As such, we next sought to use these resources to estimate the age, and representation in extant species, of conserved RBP motifs.

**Fig. 5:**
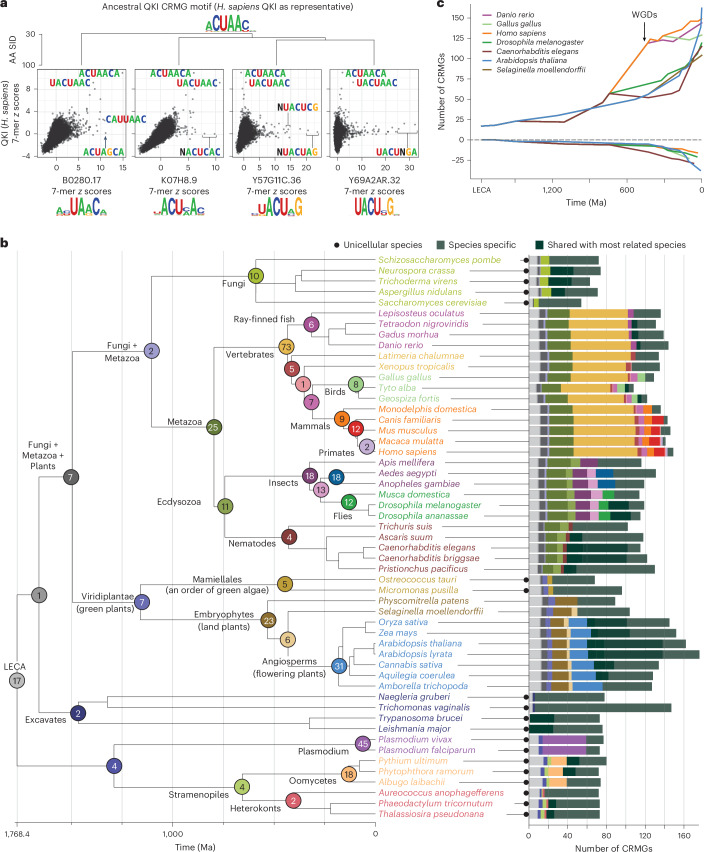
Evolution of eukaryotic CRMGs. **a**, RNAcompete 7-mer *z* scores are compared between human QKI and four homologous *C. elegans* RBPs with diverging binding specificities. The top 7-mers are labeled. The dendrogram displays the AA SID between the *C. elegans* protein RBRs. Their RNAcompete motifs are displayed below. **b**, Phylogeny of 53 species with branch points of major clades labeled (left). Branch points display the count of CRMGs gained in the shared ancestor. Species names are colored according to their major recent clade, with unicellular species indicated. The stacked bar plot (right) displays the count of extant CRMGs per species separated by ancestral origin, with colors matching branch point colors. **c**, The net number of CRMGs within the common ancestor at different time points is displayed for seven species along with the cumulative number of CRMG losses (below zero). The timing of the 2 whole-genome duplications (WGDs) that coincide with the gain of 73 CRMGs in vertebrates is indicated. Ma, million years ago; LECA, last eukaryotic common ancestor.

**Extended Data Fig. 6:**
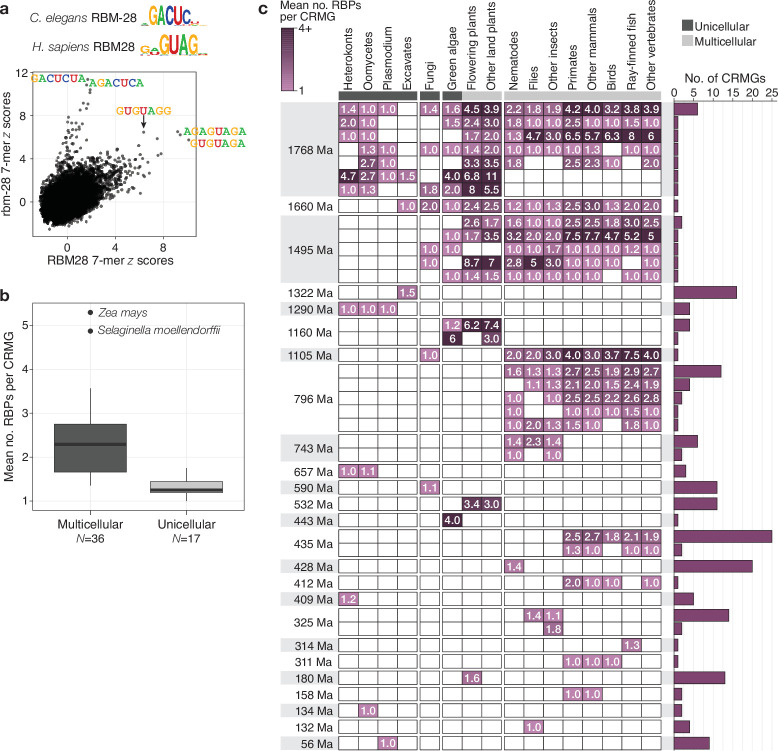
Evolution of eukaryotic CRMGs. **a**, RNAcompete 7-mer *z* scores are compared between human RBM28 and *C. elegans* ortholog RBM-28. RNAcompete motifs are shown above the plot, with some top 7-mers directly labelled. **b**, Mean number of RBPs contained within a CRMG for multicellular and unicellular species. Only multi-species CRMGs containing an RBP with an e-dist of <0.2 to an RNAcompete-measured RBP were used for the calculations. Boxes span the interquartile range (IQR) with the center line marking the median. Whiskers span from minimum to maximum value within IQR*3/2 from box boundaries. Outliers are displayed as dots and are labelled. **c**, The number of gained CRMGs at different time points are broken down by the major eukaryotic clades to which they belong. Cells in the heatmap are filled to indicate the presence, in a given clade, of the CRMGs in the barplot to the right. The mean number of RBPs within the associated set of CRMGs in extant species belonging to the associated clade is displayed in each cell. The barplot on the right displays the number of CRMGs shared between the indicated clades that arose at the given time point. As in **b**, only multi-species CRMGs containing an RBP with an e-dist of <0.2 to an RNAcompete-measured RBP are shown; Ma, million years ago.

RBP motifs are thought to be highly constrained and, as such, that new motifs are gained mainly via duplication and neofunctionalization, that is, one of the duplicated proteins retains the ancestral motif, whereas the other is released from evolutionary constraint. For example, there are at least six QKI homologs in *C. elegans*. Two homologs, GLD-1 and ASD-2, share a motif with human QKI, whereas the other four, each measured by RNAcompete, display subtle variations on the QKI motif (Fig. 5a). These four QKI homologs are clear cases of duplication and neofunctionalization; they have different measured motifs while coexisting with ASD-2 and GLD-1, which retain the ancestral QKI motif. However, there are also motifs for which there is no clear evidence for duplication and neofunctionalization; human RBM28 and *C. elegans* RBM-28 are labeled as one-to-one orthologs in UniProt but have distinct motifs (Extended Data Fig. 6a). Notably, neither has other close homologs in either organism, strongly suggesting lineage-specific specialization. Therefore, traditional sequence- and gene-tree-based measures of identifying orthologs and shared RBP function do not necessarily identify whether they have a conserved motif.

To overcome this challenge, we made use of JPLE embeddings, along with traditional methods of identifying orthology, to determine groups of proteins that share a conserved motif. We used parsimony to determine when the motif first appeared and then used the age of motifs and their presence in extant species to investigate the large-scale patterns of evolution of eukaryotic RNA motifs.

We selected 53 species that cover the evolutionary space between and within eukaryotic clades and placed all 8,957 RRM- and/or KH-domain RBPs from these species into conserved RNA motif groups (CRMGs; Supplementary Table 5). To generate CRMGs, we used e-dist to identify groups of homologous RBPs that have similar motifs or are within the same e-dist threshold of such groups using the following criteria: (1) each RBP is grouped with the RBPs to which it has the highest pairwise AA SID, (2) all RBPs within a CRMG have an observed or predicted RNA-binding profile with a PCC of >0.6 among one another, and (3) CRMGs are consistent with extant species phylogeny (Methods). The final set of 2,568 CRMGs includes only 831 CRMGs containing two or more RBPs, 82 of which contain more than 20 RBPs. These outcomes are consistent with the rapid evolution of RNA-binding specificities facilitated by the RRM and KH domains and provide nearly 1,000 CRMGs that can be analyzed in terms of species distribution and evolutionary origin.

The multiprotein CRMGs often span distantly related species, indicating that they have ancient origins. To study these origins, we identify the clade and associated ancestral node for each clade by identifying the most recent common ancestor of all extant species with RBPs in the CRMG. Figure 5b shows the number of CRMGs in each extant species, the reconstructed ancestral origins of those CRMGs and, for each ancestor node, the number of CRMGs assigned to it (Supplementary Table 6).

Several observations emerged from this analysis. First, the size of the RNA motif ‘vocabulary’ differs considerably between single- and multicellular organisms. Most single-cell organisms in Fig. 5b had between 60 and 80 CRMGs, whereas almost all multicellular organisms had more than 100, and, except for some birds, all vertebrates and flowering plants had more than 125 CRMGs per species. There are some exceptions: *Trichomonas vaginalis*, which has the largest genome among the unicellular organisms in our analysis^40,41^, had 150 CRMGs among its 183 RRM- and KH-domain RBPs, and *Physcomitrella patens*, a moss, only had 89 CRMGs. Additionally, the genomes of multicellular organisms tend to have more paralogous RBPs in the same CRMG, supporting cell-type-specific functions^42^, whereas unicellular organisms generally only have one RBP per CRMG (Extended Data Fig. 6b,c). A possible confounding factor is that the coverage of RBPs in unicellular lineages is typically lower than it is for multicellular branches. The difference in CRMGs was present even in *T. brucei*, *L. major*, *Plasmodium vivax* and *P. falciparum*, however, where we have measured or reconstructed motifs for 20–30% of the RRM- or KH-containing RBPs (coverage that rivals most metazoans), indicating that coverage is not driving this observation. Thus, the increased numbers of RBPs in multicellular genomes likely reflect not only cell-type-specific functions of RBPs binding the same motif but also a larger motif vocabulary.

Second, some CRMGs have very ancient origins. Seventeen CRMGs have the last eukaryotic common ancestor as their ancestral origin, and 19 CRMGs are present in both plants and metazoa (Supplementary Table 6). These 19 near-universal CRMGs include those representing well-studied orthologous groups of splicing factors in humans (SRSF1, SRSF2, SNRPA, SNRNP70 (with SNRNP35) and SF3B6), and CRMGs for each of the nuclear and cytoplasmic poly(A)-binding proteins, PABCP1 and PABPN1. Other near-universal CRMGs contain well-known multifunctional human RBPs; for example, one CRMG contains CELF1 through CELF6 (also known as BRUNOL or CUGBP proteins). RBPs that are members of protein complexes underlying other key post-transcriptional regulatory functions (for example, mRNA deadenylation by CCR-NOT4 (CNOT4), ribosome assembly (KRR1), the exon junction complex (RBM8A), and the cleavage and polyadenylation complex (CSTF2)) represent another group of near-universal CRMGs. The age of these CRMGs indicates that these highly conserved regulatory processes are controlled by conserved RNA motifs.

We also identified several historic periods of rapid motif gain. Figure 5c tracks the loss and net gain of CRMGs along specific lineages. The most prominent period of motif growth established 73 new CRMGs and coincided with 2 whole-genome duplications that occurred between the metazoan and vertebrate ancestors^43^. Among these vertebrate-specific CRMGs are those containing human RBPs HNRNPD, SYNCRIP and SRSF10. More modest growth in CRMGs occurred between the metazoan ancestor and the shared ancestor with fungi, when 25 new CRMGs were established. Human RBPs in these metazoan-specific CRMGs include PTBP1, QKI and MSI1. Large motif gains also occurred in the last 200 million years in 2 clades: nematodes (Nematoda; for example, *C. elegans*) and flowering plants (Angiospermae; for example, *A. thaliana*; Fig. 5b,c). In both, more than half of the CRMGs in their extant species were established in the last 200 million years, in contrast to more modest, recent CRMG gains in vertebrates, for example (Fig. 5b,c).

In nematodes, net growth in CRMGs appears to be due to rapid divergence of motifs in all nematodes (that is, separately in different nematode lineages), coupled with continuous loss of motifs from the metazoan ancestor (Fig. 5c). Such rapid rewriting and motif gain may reflect an exceptionally high spontaneous rate of gene duplications in, for example, *C. elegans*^44,45^. Consistent with our earlier observations, QKI homologs GLD-1 and ASD-2 are members of the QKI CRMG, whereas the other four homologs are members of four separate CRMGs. In each of the four cases, the ancestral species of their CRMGs is the *Caenorhabditis* ancestor, and each CRMG has the highest pairwise sequence homology with the QKI CRMG, among all other CRMGs.

In Angiospermae, the rapid gains come at the end of a continuous, accelerating gain of motifs. In this lineage, there has been continuous net growth in motif vocabularies from the ancestor of algae and land plants (23 new CRMGs) and from the land plant ancestor to flowering plants (31 new CRMGs). These events occurred with extremely rapid net growth since the Angiospermae ancestor, coupled with a relatively high rate of motif loss. *A. thaliana*, for example, lost 27 CRMGs and gained 102 new CRMGs (Fig. 5c), suggesting both rapid expansion and rewriting of the post-transcriptional *trans*-regulatory network^46^.

### Predicting stability regulators using reconstructed motifs

Taking advantage of these new sequence specificities in plant RBPs, we investigated their functions in post-transcriptional regulation. With the new repertoire of 101 RBPs with assigned motifs in *A. thaliana*, we predicted likely regulators of mRNA stability by comparing these motifs to known stability-associated *k*-mers^47^. Narsai et al. identified *cis*-regulatory elements related to mRNA decay by associating the presence of individual RNA 6-mers in mRNA 3′ untranslated regions with transcript half-lives in an *A. thaliana* cell line^47^. For each *A. thaliana* RBP with a directly measured or JPLE-reconstructed RNA-binding specificity, we identified the 6-mer (and corresponding half-life score) with the highest mean *z* score across the corresponding measured, or reconstructed, 7-mers (Supplementary Table 7).

Notably, one-fifth of the RBPs recognize three U-rich 6-mers strongly associated with mRNA destabilization: UUUUUG is recognized by TEL1 and TEL2; AUUUUG is recognized by ML1, ML4 and ML5; and UUUUUU is recognized by 16 different RBPs, the vast majority of which are known oligouridylate binders (Supplementary Table 7). An additional 12 RBPs recognize 6-mers that are strongly associated with mRNA stabilization; AAUAAG is recognized by 6 homologous CID proteins, and UGUGUG is recognized by 6 different RBPs, 5 of which have only systematic names and ARP1. ARP1 was previously identified as a sequence-specific regulator of RNA metabolism that functions in seed germination under ABA and other stress conditions; however, the sequence specificity was not determined^48^. With the JPLE-reconstructed motifs, the cellular roles of RBPs, such as ARP1, can be more readily investigated.

The CID homologs (CID8, CID9, CID10, CID11, CID12 and CID13) present a mechanism for mRNA stabilization by AAUAAG 6-mers, which represents the seventh most strongly enriched 6-mer in transcripts with long half-lives^47^. In eukaryotic cells, the timely degradation of almost the entire transcriptome is controlled by enzymatic shortening of mRNA poly(A) tails, that is, deadenylation, via the PAN2–PAN3 and CCR4–NOT complexes^49–51^. Cytoplasmic poly(A)-binding protein, PABPC1, is highly abundant and can impact the shortening of poly(A) tails by the deadenylation machinery^49,50^. Because CID RBPs (CID8, CID9, CID10, CID11, CID12 and CID13) contain a domain predicted to bind the C-terminal domain of PABPC1, we hypothesized that one consequence of this direct interaction might be the stabilization of PABPC1 on poly(A) tails, which would, in turn, modulate deadenylation.

**Extended Data Fig. 7:**
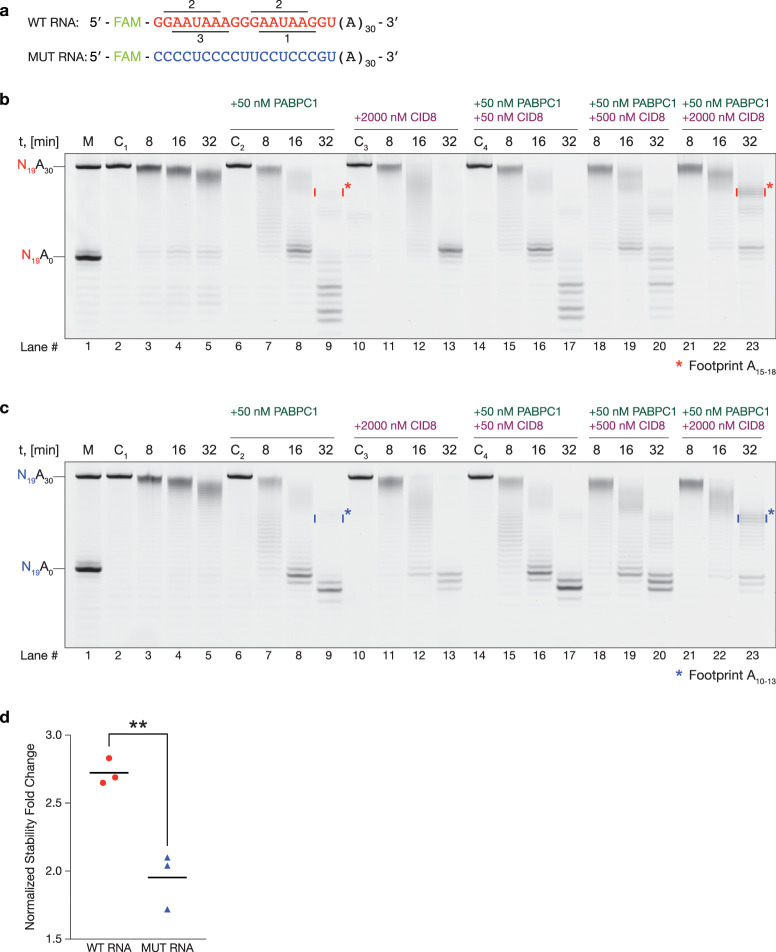
Deadenylation assay with CID8/PABPC1. **a**, Wild-type (WT) and mutant (MUT) sequences of the synthetic 5′-fluorescein-labeled RNAs. The WT sequence contains the three highest scoring 6-mers identified by JPLE for CID8, labelled 1, 2, and 3. **b** and **c**, TBE-Urea acrylamide gels of deadenylation assays with the WT (**b**) and MUT (**c**) RNAs. Lane 1 (M) shows bands for each RNA sequence with and without a poly-A tail of length 30. Control lanes (C1, C2, C3, and C4) do not contain deadenylases. Measured regions, that is, footprints, are denoted with red and blue asterisks, respectively. **d**, Grouped graph compares the normalized stability fold change of PABPC1 on WT and MUT RNAs on addition of CID8 (unpaired two-sided t-test; p = 0.004). Horizontal bars mark the mean for each group.

To test this idea and specifically examine the effect of CID RBPs on AAUAAG-containing RNA, we performed an in vitro RNA deadenylation assay using a fully reconstituted system with recombinant, purified proteins^52,53^. Fluorescently labeled RNA probes either bearing an AAUAAG 6-mer (wild-type (WT)) or a mutant (MUT) C-rich RNA (Extended Data Fig. 7a) were incubated with PABPC1 and/or CID8, followed by the addition of the NOT6–NOT7 deadenylase heterodimer to initiate deadenylation. We then monitored the extent of RNA deadenylation over time by resolving the products and intermediates by denaturing urea–PAGE at single-nucleotide resolution (Extended Data Fig. 7b,c). Both PABPC1 and CID8 individually stimulated deadenylation of both WT and MUT RNAs (Extended Data Fig. 7b,c, lanes 7–9 and 11–13 versus lanes 3–5). However, the WT RNA containing the AAUAAG 6-mer exhibited increased stability compared to the MUT, as revealed by an overall reduced rate of deadenylation (Extended Data Fig. 7b,c, lanes 19–20 and 22–23) with an accumulation of a deadenylation intermediate with a longer poly(A) tail for WT versus MUT substrate (marked with an asterisk; Extended Data Fig. 7b,c, lane 9 versus lane 23) with significant fold change (Extended Data Fig. 7d). These findings indicate that CID8, in cooperation with PABPC1, can stabilize AAUAAG-containing RNAs, directly validating the predicted role of CID8 in mRNA stability.

## Discussion

The EuPRI resource, containing the JPLE predictions, and motif data from other large-scale studies are consolidated in the CisBP-RNA web server (https://cisbp.org/rna). This resource will be broadly useful in the study of RBPs, inference of post-transcriptional regulatory networks and prediction of the functional impact of mutations in RBPs and their targets. Additionally, the EuPRI resource is qualitatively different from previous datasets. For example, Dominguez and colleagues^16^ reported a strong bias toward low-complexity motifs among human RBPs, with nearly one-third binding to homopolymeric sequences. The proportion of homopolymeric motifs is lower in the EuPRI dataset and the diversity in RBP motifs is considerably higher, particularly among RRM domain-containing proteins. These differences may reflect differing *cis*-regulatory motifs across eukaryotic clades or may be due to the data collection strategy; we focused on assaying widely conserved, phylogenetically diverse RBPs, which we suspect are more likely to have distinct motifs.

JPLE reconstructs thousands of RBP motifs using a simple, fast and easily interpretable technique. JPLE runs in seconds on a commodity laptop; this is a notable contrast with protein language models (PLMs) based on complex, deep neural network architectures that require specialized hardware. Moreover, RNA specificity-determining residues can be predicted based only on the peptide and RNA 7-mer associations captured by JPLE, suggesting that EuPRI data could be useful for fine-tuning PLMs by providing better features for modeling RBP–RNA complexes. JPLE can be easily expanded to other RBDs, if sufficient in vitro RNA-binding selection data were available, or to similarly large classes of sequence-specific DNA-binding proteins. Possible extensions to JPLE include using features from pretrained PLMs as input or using a nonlinear autoencoder or a sparse Bayesian linear factor model^54,55^.

Due to our intentionally broad survey, many of the reconstructed motifs are derived from a small number of experimentally profiled RBPs. All JPLE-reconstructed motifs have at least one profiled RBP within the stringent e-dist cutoff; depending on the eukaryotic clade, between 35% and 61% of JPLE-reconstructed motifs have multiple RBPs within this cutoff. Reconstructed motifs can also depend on RBPs outside of the cutoff; overall, JPLE assigns weights of >10% to multiple RBP motifs for between 78% and 87% of RBPs in major eukaryotic clades. Users can assess the quality of a reconstruction based on the EuPRI-provided e-dist, which, unlike AA SID, is a validated and quantitative estimate of the similarity of the motifs of two RBPs. Nevertheless, motifs assigned by homology (whether by orthology, AA SID or JPLE) are predictions and may require further experimental validation.

The RNAcompete data illustrate the flexibility of RBP sequence specificity, particularly for the RRM domain. Among these RBPs, RRM proteins collectively show statistically significant binding to nearly half of the possible 7-mers (Extended Data Fig. 1c). KH-domain RBPs recognize fewer RNA 7-mers, and although there are fewer KH domains across the eukaryotes (and fewer measured by RNAcompete than RRM domains), these trends are consistent with the flexible binding surface of the RRM domain supporting a greater degree of evolutionary innovation in recognized target sequences than the binding cleft used by the KH domains^26,56^. Notably, 54.3% (143 of 263) of human RRM and KH RBPs have motifs that are younger than the metazoan ancestor; that is, most human RBPs do not have the same RNA motif as their closest fly or worm homolog. This observation alone underscores the importance of studies of the evolution of post-transcriptional regulatory networks.

## Methods

### Identifying eukaryotic RBPs

To identify RBPs across 690 eukaryotic organisms, we scanned protein sequences for well-characterized RBDs using HMMER^57^ with recommended parameter settings (‘full sequence *E* value’ (sequence_eval) ≤ 0.01 and ‘domain conditional *E* value’ (c-Evalue) ≤ 0.01). We used the RBD profile HMMs (pHMMs) CSD, KH_1, La, NHL, PUF, S1, SAM_1, YTH, zf-CCCH, zf-CCHC, zf-CCHH and zf-RanBP from the Pfam database^58^. For RRMs, we defined a new, longer pHMM as the Pfam RRM_1 pHMM does not include all residues that contact RNA. This extended RRM pHMM is based on PDB RRM structures, and its construction is described in Supplementary Note 1.

We grouped all identified RBPs into ‘RBP families’ by their domain architecture, that is, the type, number and order of the RBDs in their protein sequence (for example, RRM, RRM-RRM, KH-RRM and RRM-RRM-KH).

### Calculating amino acid sequence identities

We used two separate methodologies for aligning the RBRs of RBPs and calculating their pairwise AA SIDs. The first method, ‘RBP family-wise AA SID calculations’, was used for calculating AA SIDs within RBP families, where all RBPs have the same RBD architecture. We generated an RBR sequence for each protein by concatenating its individual RBD sequences in order and applied clustalOmega^59^ with default settings to generate multiple sequence alignment for all RBR sequences within an RBP family. We defined the AA SID between each pair of RBRs in an RBP family as the proportion of exactly matched, aligned residues in the multiple sequence alignment.

We used a second method for individual pairs of RBPs, ‘RBP pairwise AA SID calculations’, to allow for comparisons between RBPs in different RBP families. Here, we defined the RBRs as the subsequence of the RBP that contains all of its RBDs plus up to 15 flanking amino acids before and after each RBD in the RBR. These flanks were added to include linker regions and C and N termini. To align two RBRs, we aligned each RBD (with flanks) in one RBR to each RBD in the other RBR using BLOSUM62 scoring (gap opening –11, gap extension –1; Needleman–Wunsch). For each RBD-to-RBD alignment, we computed the AA SID (number of exact aligned matches / total length). If the two RBRs had the same number of RBDs (for example, RRM1–RRM2 and RRM1–RRM2), we calculated the pairwise RBR AA SID as the mean AA SID of each pair of corresponding RRMs (for example, mean (RRM1 versus RRM1 AA SID, RRM2 versus RRM2 AA SID)). If the two RBRs had differing numbers of RBDs (for example, RRM1–RRM2 and RRM1–RRM2–RRM3), we computed the mean AA SID of all possible alignments of the shorter RBR to the longer RBR, only allowing alignments where adjacent RBDs are aligned (for example, RRM1–RRM2 can only be aligned to RRM1–RRM2 or RRM2–RRM3 of a three-RRM RBR). The maximum AA SID of the shorter RBR alignments is used as the pairwise RBR AA SID. This procedure is designed to account for duplication and deletion events during evolution of protein sequences.

To infer the RNA-binding specificity of an uncharacterized protein based on AA SID (for example, the ‘70% rule’), we identified the RNAcompete-measured RBP with the highest overall AA SID and used the RNAcompete RNA-binding profile of this protein as the RNA-binding profile for the uncharacterized protein. The AA SID between the two proteins was used as the confidence score for this prediction.

### Selecting RBPs for RNAcompete

To select eukaryotic RBPs for characterization by RNAcompete, we considered RRM-, KH- and CCCH-containing RBPs from 45 well-annotated eukaryotes, representing model organisms and diverse species across the eukaryotic tree^31^, identified as described in ‘Identifying eukaryotic RBPs’. For this task, we used AA SIDs calculated using the RBP family-wise method. We used four different strategies (described in Supplementary Note 2) to select RBPs to maximize the utility of the dataset. Collectively, the procedure produced 277 diverse RBPs for experimental characterization using RNAcompete.

### Measuring RNA sequence specificities with RNAcompete

RBP inserts (refer to Supplementary Table 1) were commercially synthesized (Bio Basic) for all 277 selected RBPs and cloned into a custom expression vector, pTH6838, using AscI and SbfI restriction enzymes sites^32^. Glutathione *S*-transferase (GST)-tagged RBPs were purified from *Escherichia coli*, and RNAcompete experiments were performed to determine their RNA-binding profiles. RNAcompete, a microarray-based in vitro RNA-binding assay, has been extensively detailed elsewhere^17,32^ and in Supplementary Note 3. Briefly, recombinant GST-tagged RBPs are incubated with ~241,000 designed (not randomized) RNA probes, each about 40 nucleotides long. RNA probes are designed to possess low probabilities for base pairing (that is, they are single stranded) and to represent each 7-mer at least 310 times. RBP–RNA complexes are affinity purified, and bound RNAs are extracted and labeled with Cy3 or Cy5. Abundance is measured on a custom Agilent 244K microarray.

Measured probe intensities were centered, and their variance was normalized for each protein (columns) and RNA probe (rows) to control for variation in RNA and protein concentrations. Next, a score was robustly computed for each 7-mer as the mean probe intensities of the inner 95% of probes that contain the 7-mer (the probes with the highest and lowest intensities were excluded). These 7-mer scores were transformed to *z* scores, using the mean and standard deviation of all 7-mer scores. The vector of *z* scores for 16,382 7-mers is referred to as the RNA-binding profile; two 7-mers (GCTCTTC and CGAGAAG) were removed because they correspond to the SapI/BspQI restriction site. For visualization purposes, position weight matrices were generated by aligning the ten most enriched 7-mers without allowing for gaps. A 7-mer sequence is considered to be ‘specifically bound’ by an RBP if its Benjamini–Hochberg-adjusted *z*-score *q* value is <0.01.

We evaluated RNAcompete experiments for the 277 selected RBPs according to previously described success criteria^32^ and identified a subset of 174 RBPs with successful RNAcompete experiments. Combining these experiments with those from a previous study^17^, we generated a set of 420 experiments covering 379 unique RBPs (across 381 unique constructs). For RBPs with more than one RNAcompete experiment, we calculated the mean of the *z* scores for the top ten 7-mers and used the experiment with the highest mean in subsequent analyses (Supplementary Table 1).

We additionally assessed variability in the 7-mer *z* scores for all 420 experiments using a bootstrap analysis. For each RNAcompete experiment, we sampled probes with replacement 100 times and recalculated the *z* scores. The mean and standard deviation for each 7-mer *z* score from the bootstrap samples are available at https://hugheslab.ccbr.utoronto.ca/supplementary-data/RBPZoo/.

### JPLE

In JPLE, each RBP is assigned a fixed-length peptide profile vector **p**′, which contains the count of all 5-mer peptide templates (with four specified amino acids and one wildcard character, which cannot be in the first or fifth position) within any of its RBDs, including 15 flanking nucleotides on either side of each RBD (Fig. 2a). This ‘bag-of-peptides’ representation has been used previously for a number of protein function prediction tasks, including homology modeling^28,60^. Each RBP is also associated with a fixed-length RNA-binding profile vector **r**′ composed of its RNAcompete *z* scores for all RNA 7-mers (Fig. 2b), apart from GCUCUUC and CGAGAAG, which are target sequences of the restriction enzymes used in the assay. The row vectors **p**′ and **r**′ for the *n* = 355 training set RBPs were stacked to form matrices *P*′ ∈ ℝ^*n* × *p*′^ and *R*′ ∈ ℝ^*n* × *r*′^, where *p*′ is the number of RBP 5-mers, and *r*′ is the number of RNA 7-mers. Each element of the matrix was divided by the Euclidean norm of its row, and each column was centered by subtracting the corresponding column mean (collected in vectors **μ**_*P*_ and **μ**_*R*_ for *P*′ and *R*′, respectively) from each element. We then removed columns with zero variance from both matrices. The final transformed matrices, *P* ∈ ℝ^*n* × *p*^ and *R* ∈ ℝ^*n* × *r*^, have *p* and *r* columns, respectively, and consist of 355 stacked row vectors **p** and **r**, respectively.

The matrices *P* and *R* were concatenated column-wise to form the joint protein representation [*PR*] ∈ ℝ^*n* *×* (*p* *+* *r*)^ (Extended Data Fig. 2a), to which singular value decomposition (SVD) was applied, giving

$$[{\it{P}}{\it{R}}]={\it{U}}{\varSigma}{{\it{V}}}\,^{\mathrm{T}}$$

where *U* ∈ ℝ^*n* *×* rank([*PR*])^, *Σ* ∈ ℝ^rank([*PR*]) *×* rank([*PR*])^, *V* ∈ ℝ^(*p* *+* *r*) *×* rank([*PR*])^ and T means transpose. The diagonal entries *Σ*_*ii*_ of *Σ* are singular values *σ*_*i*_, whereas the columns of *U* and *V* (that is, **u**_*i*_, **v**_*i*_) form the orthonormal basis of [*PR*] (*U*^T^*U* = *V*^T^*V* = *I*_rank([*PR*])_). Typically, when performing dimensionality reduction using SVD, the lowest *σ*_*i*_ are set to 0, effectively removing basis vectors **u**_*i*_ and **v**_*i*_ that explain the least variance in [*PR*]. In JPLE, however, we retain basis vectors contributing the most to the variance of *R* without regard to how much of the variance of *P* is explained. To compute the per-basis-vector contribution, we expressed the variance of *R* in [*PR*] as

$$\begin{array}{l}{\rm{Var}}({\it{R}})={\rm{tr}}({{\it{RR}}}^{{\rm{T}}})={\rm{tr}}({{\it{R}}}^{{\rm{T}}}{\it{R}})={\rm{tr}}({{\it{V}}}_{{\it{R}}}{\varSigma}\,{{\it{U}}}\,^{\rm{T}}{\it{U}}{\varSigma}{{\it{V}}_{\it{R}}}^{\rm{T}})\\={\rm{tr}}({{\it{V}}}_{{\it{R}}}{\varSigma}\,^{2}{{\it{V}}_{\it{R}}}^{\rm{T}})=\mathop{\sum}\limits_{i}^{{\rm{rank}}([{\it{PR}}])}{{\rm{\sigma }}_{i}}^{2}{{\mathbf{v}}_{\it{R},{\it{i}}}}^{\rm{T}}{{\mathbf{v}}_{\it{R},{\it{i}}}}\end{array}$$

where *V*_*R*_ are the columns of *V* that correspond to those of *R* in [*PR*]. The top *d* basis vectors and singular values, ranked by their variance contribution to *R* in [*PR*], were retained. By performing SVD on *R* alone for the 355 training set proteins, we determined that 122 singular vectors were required to achieve a minimum Pearson correlation coefficient of 0.95 between their reconstructed (r*) and measured (**r**′) RNA-binding profiles (Extended Data Fig. 2b). These selected singular vectors explain 96% of the variance in both *R* alone (Extended Data Fig. 2b) and *R* in [*PR*] (Extended Data Fig. 2d), demonstrating that *d* = 122 is sufficient to capture most of the variance of *R* in [*PR*]. With this, the original SVD formulation can be written as the following approximation:

$${\mathbf{[}}{\it{PR}}{\mathbf{]}}\approx {{\it{U}}^{\prime}{\varSigma}^{\prime}{\it{V}}^{{\prime}{\mathrm{T}}}}$$

where *U*′ ∈ ℝ^*n* × *d*^, *Σ*′ ∈ ℝ^*d* × *d*^ and *V*′ ∈ ℝ^(*p* *+* *r*) × *d*^. The rows of the matrix *W* = *U*′*Σ*′ ∈ ℝ^*n* × *d*^ each represent a latent embedding of one of the training set RBPs in the subspace spanned by the columns of *V*′.

### Protein query

In a protein query, JPLE maps a peptide profile **p** to its reconstructed RNA-binding profile r* (Fig. 2c and Extended Data Fig. 2c). Given an RBP with an uncharacterized RNA specificity, JPLE computes its latent embedding **w**_u_ by deconvolving its peptide profile **p**_u_ according to a mixture matrix containing the orthonormal bases in *V*_*P*_′ (columns of *V*′ that correspond to those of *P* in [*PR*]), that is,

$${{\mathbf{p}}}_{{\rm{u}}}={{\it{V}}_{\it{P}}}^{\prime}{{\mathbf{w}}}_{{\rm{u}}}$$

The equation above can be solved to find the best least squares estimate **w**_u_* of the embedding that reproduces **p**_u_ using the pseudoinverse of *V*_*P*_′:

$${{\mathbf{w}}_{\rm{u}}}^{*}={\left({{\it{V}}_{\it{P}}}^{{\prime}{\rm{T}}}{{\it{V}}_{\it{P}}}^{\prime}\right)}^{-{1}}{{\it{V}}_{\it{P}}}^{{\prime}{\rm{T}}}{{\mathbf{p}}}_{{\rm{u}}}$$

This embedding **w**_u_* can be associated with a reconstructed RNA-binding profile **r**_u_* using one of two different approaches. The first approach, termed global decoding, uses a linear mapping and is not used for protein queries, but an equivalent method is used for RNA queries and is described in the next section. The second approach, termed local decoding, is used for protein queries and reconstructs the RNA-binding profile based on training set RBPs with nearby embeddings, thereby implementing a nonlinear mapping. To do so, we first define the e-dist *ε*_u*i*_ between an uncharacterized and training set RBP to be the cosine distance between their latent embeddings, that is,

$${\varepsilon }_{{\rm{u}}{i}}=1-{{\mathbf{w}}_{\rm{u}}}^{*{\rm{T}} }{{\mathbf{w}}}_{{i}}/{\rm{||}}{{\mathbf{w}}_{\rm{u}}}^{*}{\rm{||}}\;{\rm{||}}{{\mathbf{w}}}_{{i}}{\rm{||}}$$

where **w**_*i*_ (*i*th row of *W* as a column vector) is the latent embedding of the training set RBP_*i*_. The e-dist around each uncharacterized RBP_u_ was used with a radial basis function kernel (0 mean and *γ* = 25) to obtain the e-sim *s*_u__*i*_:

$${s}_{{\rm{u}}{i}}=\exp (-\gamma {{\varepsilon }_{{\rm{u}}{i}}}^{2})$$

For each uncharacterized RBP_u_, we constructed a set of *N* neighborhood training set proteins as follows, *N* = {*i* | *s*_u__*i*_ ≥ 0.01}. The reconstructed RNA-binding profile **r**_u_* of the uncharacterized RBP_u_ was computed as the average RNA-binding profile **r**_*i*_ of all neighborhood training set proteins, weighted by their e-sim *s*_u__*i*_:

$${{\mathbf{r}}_{\rm{u}}}^{{*}}=(\sum_i{s}_{{\rm{u}}{i}}{{\mathbf{r}}}_{{\it{i}}})/(\sum_i{s}_{{\rm{u}}{i}})\forall i\in N$$

If | *N* | = 0, **r**_u_* is set to the RNA-binding profile of the training set protein with the highest e-sim. Overall, local decoding increases the impact of training set proteins with similar embeddings on the reconstruction while negating the impact of training set RBPs with more dissimilar embeddings.

### RNA query

In an RNA query, JPLE maps an RNA-binding profile **r** to its reconstructed peptide profile p* (Fig. 2c and Extended Data Fig. 2c) using the global decoding approach. Although the preserved basis vectors in *V*′ account for 96% of the variance in the RNA specificities *R*, they only explain 44% of the variance in the protein peptide profiles *P* (Extended Data Fig. 2d). Thus, they are correlated with at least one of the RNA specificities, thereby characterizing functionally important peptides for RNA recognition.

To improve the representation of peptide sequences in the training set RBPs, we used a ‘data augmentation’ approach to train a version of JPLE for RNA queries. Specifically, we augmented the training set with RBPs that have high homology to those in the training set. These homologs lack RNAcompete measurements but likely have similar binding specificities to those measured by RNAcompete. This data augmentation strategy improved RNA queries but did not have a clear impact on protein queries, so it was not used for the version of JPLE trained for protein queries.

The added homologous RBPs were identified by using HMMER^57^ (*E* value of ≤1 × 10^−15^) to align RBP sequences containing RRM or KH domains to those with measured RNA specificities. Those uncharacterized RBPs with AA SIDs ranging from 50% to 99% to any measured RBPs were considered hits (that is, homologs). For a given training set RBP, its 5-mer counts were computed across the alignments of all hits. The resulting peptide counts, normalized by the number of distinct peptides at a given position, were used as a protein representation vector **p**_+_ for the given training set RBP. These augmented representations *P*_+_ were then concatenated with the measured RNA sequence specificities *R*_+_ to train JPLE, resulting in their latent embeddings *W*_+_ and orthonormal bases *V*_+_′.

Like a protein query, query RNA profiles were embedded into the latent space *W*_+_. However, the latent embedding **w**_+u_ of the query profile was obtained by deconvolving its RNA profile **r**_u_ using the mixture matrix *V*_+__*R*_′:

$${{\mathbf{r}}}_{{\rm{u}}}={{\it{V}}_{+{\it{R}}}}^{{\prime}}{{\mathbf{w}}}_{+{\rm{u}}}$$

One can solve the equation above to compute an estimate, **w**_+u_*, of the latent embedding by multiplying both sides by the pseudoinverse of *V*_+*R*_′, giving

$${{\mathbf{w}}_{+{\rm{u}}}}^{{*}}={({{\it{V}}_{+{\it{R}}}}^{{\prime}\rm{T}}{{\it{V}}_{+{\it{R}}}}^{{\prime}})}^{-{{1}}}{{\it{V}}_{+{\it{R}}}}^{{\prime}\rm{T}}{{\mathbf{r}}}_{{\rm{u}}}$$

After computing this estimate, we used global decoding to map the estimated latent embedding **w**_+u_* to its reconstructed peptide profile **p**_u_*, representing the relative contribution of each peptide 5-mer to the input RNA specificities, as follows:

$${{\mathbf{p}}_{\rm{u}}^{\;{*}}}={{\it{V}}_{+{\it{P}}}}^{{\prime}}{{\mathbf{w}}_{+{\rm{u}}}}^{{*}}+{\mathbf{\upmu}}_{{\it{P}}}$$

Thus,

$${{\mathbf{p}}_{\rm{u}}}^{{*}}={{\it{V}}_{+{\it{P}}}}^{{\prime}}{({{\it{V}}_{+{\it{R}}}}^{{\prime}\rm{T}}{{\it{V}}_{+{\it{R}}}}^{{\prime}})}^{-{1}}{{\it{V}}_{+{\it{R}}}}^{{\prime}\rm{T}}{{\mathbf{r}}}_{{\rm{u}}}+{{\mathbf{\upmu }}}_{{\it{P}}}$$

Notably **p**_u_* is a linear function of **r**_u_, but the above model corresponds neither to ordinary linear regression (where, among other differences, *V*_+*R*_′ and *V*_+*P*_′ would be replaced with *R*_+_ and *P*_+_, respectively) nor to principal components regression, which would essentially correspond to using an embedding of **r**_u_ to predict **p**_u_ directly rather than **w**_+u_*, the embedding of **p**_u_. This is an important difference because **w**_+u_* only retains information about **p**_u_ relevant to its RNA sequence specificity.

### Alternative JPLE implementations and RNA-binding specificity prediction methods

#### Protein sequence representation models

We retrieved pretrained ‘Unirep’ and ‘Transformer’ (also referred to as ‘bert’ in the code repository) models reimplemented and trained as part of the tasks assessing protein embeddings (TAPE) framework^33^ and embedded the RBR sequences for the 355 training set RBPs using TAPE’s tape-embed command. We then used the 1,900- and 768-dimensional embeddings generated by the Unirep and Transformer models, respectively, to train two linear regression models with ridge regression (*λ* = 0.0001) to predict the RNA-binding profiles.

#### Affinity regression

Affinity regression is a conceptually similar machine learning approach designed for predicting RNA specificities of RBPs^28^. Instead of modeling the direct mapping between *P* and *R*, however, affinity regression learns the interaction, *A*, between RBP amino acid 4-mer counts *P* and RNA 5-mer counts *D* to reconstruct *R* during training,

$${{\it{DAP}}}^{{\rm{T}}}\approx {{\it{R}}}^{{\rm{T}}}$$

where *D* ∈ ℝ^*r* × *r*′^, *A* ∈ ℝ^*r*′ × *p*^, *P* ∈ ℝ^*n* × *p*^ and *R* ∈ ℝ^*n* × *r*^. Affinity regression was applied to the same set of 355 training set RBPs for direct comparison to JPLE. Supplementary Note 4 contains further information about affinity regression and our implementation of affinity regression.

#### Nearest neighbor model

For an RBP with uncharacterized RNA specificities, the nearest neighbor model computes the cosine similarity between its peptide profile and all 355 training set peptide profiles. The uncharacterized RBP adopts the RNA-binding profile of the closest training set RBP.

#### Protein–nucleic acid structure models

We evaluated the ability of RF2NA^34^ and AF3 (ref. ^35^) to differentiate between ‘binding’ and ‘nonbinding’ RBP–RNA interactions. For each of the 355 RBPs containing an RRM or KH domain, we generated both a ‘binding’ and ‘nonbinding’ set of RNA 7-mers. To do this, we ranked the 7-mers by their *z* scores and excluded the top and bottom 2.5% to account for potential artifacts. The top 50 7-mers were assigned to the ‘binding’ set, whereas the 50 7-mers closest to the median were designated as the ‘nonbinding’ set. This approach produced a total of 35,500 RBP–RNA pairs.

Subsequently, we used RF2NA to predict the three-dimensional (3D) structures of these RBP–RNA pairs. Following the RF2NA evaluation method^34^, we used the mean interface predicted aligned error (PAE) as a proxy for RF2NA’s predicted binding specificity, with lower values indicating higher binding specificities. For each RBP–RNA residue pair, we calculated its average PAE by taking the mean of the PAE from the RBP to the RNA residue and the PAE from the RNA to the RBP residue. We then determined the mean interface PAE by averaging these values across all interface residue pairs, defined as RBP–RNA residue pairs within 4.5 Å of each other. Structures lacking any interface residue pairs were excluded from the analysis, resulting in 35,479 mean interface PAE values.

We similarly used AF3 to predict the 3D structures and used the same methodology to compute the mean interface PAE for each RBP–RNA pair. In total, 35,213 mean interface PAE values remained after excluding structures without interface residue pairs.

### PDB cocomplex structures

We retrieved entries of cocomplex structures that include both RRM-containing RBPs and RNAs from the PDB^36^. These 89 PDB entries contain a total of 156 RBP chains whose RRM domains were identified and extracted with HMMER^57^ using the standard Pfam RRM_1 profile HMM^58^. We identified 156 RRM domains, contained within 119 protein chains, with hmmsearch using default settings^57^. The RRM domain sequences were extended by 35 amino acids in both directions and merged if the sequences overlapped in the same protein chain, leading to 118 RRM-containing RBRs.

For each extracted PDB structure, we identified its interface protein residues as those with any carbon atoms within 5 Å of any RNA atom. The RBP-binding motifs, in IUPAC format, were defined as the connected RNA bases within 5 Å of an RBR carbon atom.

To assign RBPs with RNAcompete measurements to those within PDB structures, we computed pairwise AA SIDs and motif overlap. The measured RBP with the highest motif overlap (a minimum of 3.5 nucleotides) and at least 50% AA SID was selected as the best match. Given that most PDB structures have no more than two RRM domains in contact with RNA, some RNAcompete experiments were matched to multiple PDB structures. Subsequently, redundant PDB structures that share more than 70% AA SID were deduplicated by retaining the one with the longest protein sequence.

In total, 27 qualifying PDB structures were assigned a measured RBP; however, we reduced the set to 26 as one PDB structure contained only the third RRM of ELAVL1, which functions primarily as a dimerization domain^61^.

### Assignment and assessment of RISs

We conducted RNA queries on all RNAcompete experiments using JPLE with leave-one-out cross-validation and obtained their reconstructed peptide profiles **p**_u_*, which were then stacked row-wise to form the matrix P* ∈ ℝ^*n* × *p*^. The matrix was standardized by its column mean and standard deviation.

For interface prediction, RISs were calculated for each PDB entry from the reconstructed peptide profile **p**_i_* of the matched RNAcompete-measured RBP. To assign an importance score to each residue in the PDB RBR, we divided each element in **p**_i_* by its number of occurrences in the RBR, and then, for each residue, we summed the values of the overlapping peptides in **p**_i_*.

As a baseline for interface characterization, we compared JPLE’s RISs to sequence conservation, along with a random forest model trained directly on selected PDB structures. Details of these methods are in Supplementary Note 5.

### Reconstructing RNA sequence specificities for 690 eukaryotes

Using our JPLE model trained on all 355 RNAcompete-measured RBPs, we predicted RNA sequence specificities for RBPs across 690 eukaryotes. RBRs were identified across eukaryotic species as described in ‘Identifying eukaryotic RBPs’.

We used JPLE to perform protein queries on each of the ~88,000 detected RBRs with RRM and KH domains. Reconstructions with an e-dist of less than 0.127 were considered confident predictions, corresponding to an average PCC between reconstructed and measured RNA-binding profiles of 0.75 and a rolling average PCC of 0.70 at all levels of recall (Extended Data Fig. 4b). Moreover, RNA queries were conducted using the version of JPLE augmented by homologous sequence, as per above, and RISs were computed for all RBRs with confident RNA-binding profile predictions. Secondary structure profiles were computed with SCRATCH^62^ for all measured RBR sequences. RBR sequences of unmeasured RBPs were aligned to the measured RBPs, and the predicted secondary structure profile of the RBR with the highest AA SID was used for the visualization on CisBP-RNA.

### Using JPLE e-dist to determine groups of RBRs with common motifs

To study the evolution of RNA motifs in eukaryotes, we sought to use JPLE to identify groups of RBPs with a conserved RNA motif. First, we investigated the relationship between e-dist and RNA motif similarity for RBPs with low sequence homology to the closest RNAcompete-measured RBP. To do so, we used a neighbor-joining algorithm to cluster these proteins into 50 sets of RBPs with high intragroup AA SID and low intergroup AA SID. After clustering, 80% of RBPs had a maximum intergroup AA SID of less than 30%. We trained 50 different JPLEs, each with one of the 50 groups held out, and evaluated how well e-dist within the held-out group correlated with the PCC between the pairs of held-out, low-sequence-homology RBPs. We found that pairs of held-out RBPs with an e-dist of <0.2 had an average PCC of 0.62 (Extended Data Fig. 2e), the lower bound of the PCCs of technical replicates (Fig. 1c). As such, we used an e-dist threshold of 0.2, along with sequence homology, to identify clusters of uncharacterized RBPs with a shared motif and derived from the same ancestor. Note that the 0.2 e-dist cutoff is the same as that used in the main text to estimate recall of JPLE in leave-one-out cross-validation, where it is associated with a PCC of 0.748. Here, the average correlation is lower because the reconstruction of the RNA profiles of the held-out, low-homology RBPs is more challenging.

### Clustering RBPs into CRMGs and characterizing their last common ancestors

To define clusters of evolutionarily related RBRs with conserved RNA sequence specificity, we used a multistep clustering algorithm that incorporated JPLE latent distances, pairwise AA SID, a selected species tree and agglomerative clustering. We used TimeTree^63^ and the ETE Toolkit^64^ to select and extract evolutionary distances between 53 eukaryotic species that cover the evolutionary space between and within eukaryotic clades. Collectively, these species contain 8,957 RBR sequences that are exclusively composed of RRM and KH domains. We used a global alignment (BLOSUM62 and gap penalty 11, 1 (open, extension), Needleman–Wunsch) to compute pairwise AA SIDs between all pairs of RBR sequences (see ‘Calculating amino acid sequence identities’). Between RBPs with the same or one domain difference, however, we computed sequence identities using the shorter sequence length as a reference to account for homologs that lost a single domain. For all others, we used the longer sequence as a reference, assuming that orthologs generally do not gain or lose more than one domain.

We constructed a ‘highest homology network’ between pairs of RBRs in different species. The ‘highest homology network’ contains one node for each of the 8,957 RBR sequences, and we connected each RBR to the RBR with the highest AA SID in each of the other 52 species. This highest homology network is used as a scaffold for our clustering, thereby ensuring that we only combine RBR sequences likely to be descended from a common ancestral protein into CRMGs. We then remove any links in the highest homology network between RBRs with an e-dist of greater than 0.2. This created a set of 2,463 connected components of the filtered network; these components are used as the initial set of CRMGs. Each pair of RBRs in a cluster are thus connected by at least one path where each link is both a highest homology link and has an e-dist of <0.2. Examining this initial draft of the CRMGs, we identified potential false positives and further refined our set of CRMGs using the process described in Supplementary Note 6. After refinement, we had 2,568 CRMGs, each of which was assigned to a clade represented by the ancestral node in the species tree by inferring the most recent common ancestor of all extant species with RBPs in the CRMG.

### Identifying putative *A. thaliana* stability regulators and deadenylation assay

For the 101 *A. thaliana* RBPs with an RNAcompete-measured (5) or JPLE-reconstructed (96) RNA-binding profile, we calculated a score for all possible RNA 6-mers to compare to the half-life scores from Narsai et al.^47^. To calculate the score for a given 6-mer, we took the mean of the *z* scores for all 7-mers containing the 6-mer (Supplementary Table 7).

#### RNA sequences

The WT RNA sequence was designed to contain the highest-scoring 6-mer (AAUAAG) bound by the CID homologs, as well as the second- and third-highest-scoring 6-mers, GAAUAA and AAUAAA (Extended Data Fig. 7a). The C-rich MUT RNA sequence was designed not to contain high-scoring CID homolog 6-mers. Both sequences were checked to ensure that they would not form RNA secondary structures or G-quadruplexes using RNAfold^65^ and the QGRS Mapper^66^, respectively.

#### DNA constructs

NOT6 and NOT7 constructs were described previously^52,53^. The pETM11 plasmid with the gene encoding full-length (residues M1-V636) PABPC1 (UniProt ID P11940) was obtained from Addgene (146642). The gene encoding full-length CID8 (residues M1-N314; UniProt ID Q9C8M0) was cloned into an in-house vector (pnEK-NSupH) in a frame with the N-terminal His_6_-SUMO tag using the Gibson assembly method.

#### Protein production and purification

The production and purification of the NOT6–NOT7 deadenylase heterodimer was performed as previously described, with minor adjustments^52,53^. The protocol is described in detail in Supplementary Note 7.

PABPC1 was produced in BL21(DE3) Star *E. coli* cells using 4 l of LB medium at 30 °C for 5 h. To induce production, 1 mM IPTG was added after reaching an optical density at 600 nm of 0.3. The cells were collected and resuspended in a lysis buffer containing 50 mM HEPES/NaOH (pH 7.0), 1,000 mM NaCl, 5% (wt/vol) glycerol and 25 mM imidazole and lysed by sonication. The lysate was clarified by centrifugation at 40,000*g* for 45 min and loaded on a 5-ml nickel-charged IMAC column (Cytiva). The bound protein was washed with 20 column volumes of lysis buffer and eluted from the column in 1-ml fractions with the same buffer supplemented with 250 mM imidazole. Peak fractions from nickel affinity chromatography were then pooled together and incubated with TEV protease at 4 °C overnight to cleave off the His_6_ tag. The next day, PABPC1 was loaded and eluted on a Superdex 200 26/600 column (Cytiva) equilibrated in a buffer containing 50 mM HEPES/NaOH (pH 7.0), 200 mM NaCl, 5% (wt/vol) glycerol and 2 mM DTT. The peak fractions were then pooled together, concentrated to ~10 mg ml^−1^, flash-frozen in liquid nitrogen and stored at –80 °C until use.

CID8 was produced in BL21(DE3) Star *E. coli* cells using 2 l of autoinduction medium at 20 °C overnight. The cells were collected and resuspended in a lysis buffer containing 50 mM potassium phosphate (pH 7.5), 1,000 mM NaCl and 25 mM imidazole and lysed by sonication. The lysate was clarified by centrifugation at 40,000*g* for 45 min and loaded on a 5-ml nickel-charged IMAC column (Cytiva). The bound protein was washed with 20 column volumes of lysis buffer and then eluted from the column in 1-ml fractions with the same buffer supplemented with 250 mM imidazole. Peak fractions from nickel affinity chromatography were then loaded and eluted on a Superdex 200 26/600 column (Cytiva) equilibrated in a buffer containing 20 mM HEPES/NaOH (pH 7.5), 300 mM NaCl and 2 mM DTT. The peak fractions were then pooled together, concentrated to ~2 mg ml^−1^, flash-frozen in liquid nitrogen and stored at –80 °C until use.

#### Deadenylation assays and quantification

Deadenylation reactions were performed in a buffer containing 20 mM PIPES (pH 7.0), 40 mM NaCl, 10 mM KCl and 2 mM magnesium acetate at 37 °C. Assays were performed as described previously^53^ with the following modifications. To the 5′-fluorescein-labeled WT or MUT RNA substrate (50 nM; biomers.net; for sequences, see Extended Data Fig. 7a) were added PABPC1 (50 nM) and His_6_-SUMO-CID8 at 1:1, 10:1 and 40:1 ratios relative to PABPC1 and incubated on ice for 15 min. To start the reaction, 100 nM NOT6–NOT7 deadenylase heterodimer was added. To stop the reaction at the corresponding time point, 3-fold reaction volumes of RNA loading dye were added (95% (vol/vol) deionized formamide, 17.5 mM EDTA (pH 8) and 0.01% (wt/vol) bromophenol blue). The products were resolved on a denaturing TBE–urea polyacrylamide gel, which was subsequently imaged using a Sapphire FL Biomolecular Imager (Azure Biosystems).

The deadenylation products were analyzed and quantified using Azure Spot Pro (Azure Biosystems). The final metric represents the normalized stability fold change of PABPC1 footprints in the presence of CID8 on WT and MUT RNAs.

Briefly, the signal intensities of the PABPC1 footprint on WT and MUT RNAs were quantified at the 32-min time point, with and without a 40-fold excess of CID8 (Extended Data Fig. 7b,c, lanes 9 and 23). Quantification was localized within a defined region corresponding to the footprint. For each sample, the signal of the PABPC1 footprint was normalized as a fraction of the total signal intensity within the corresponding lane. The normalized fraction value for PABPC1 in the presence of CID8 was then divided by the normalized fraction value for PABPC1 alone, yielding a stabilization fold for both WT and MUT RNAs. The above steps were performed in triplicate for both WT and MUT RNAs to ensure reproducibility. Measurements and calculations are shown in Supplementary Table 8. An unpaired two-sided *t*-test was performed to compare the stabilization fold change between WT and MUT RNAs and to assess whether CID8 exerted a significantly different stabilizing effect on the two RNA species. The graph was generated using GraphPad Prism 10.

### Reporting summary

Further information on research design is available in the Nature Portfolio Reporting Summary linked to this article.

## Online content

Any methods, additional references, Nature Portfolio reporting summaries, source data, extended data, supplementary information, acknowledgements, peer review information; details of author contributions and competing interests; and statements of data and code availability are available at 10.1038/s41587-025-02733-6.

## Supplementary information

Supplementary Information Supplementary Notes 1–7.

Reporting Summary

Supplementary Tables 1–8 Supplementary Table 1. RNAcompete experimental details. Supplementary Table 2. Performance of JPLE and other RNA-specificity prediction methods for the 355 training set proteins. Supplementary Table 3. Performance of RISs and other prediction metrics for 26 PDB cocomplex structures. Supplementary Table 4. Count of identified RBPs and RBPs with assigned motifs across 690 eukaryotes. Supplementary Table 5. CRMG assignments for 8,957 RBPs from 53 species. Supplementary Table 6. CRMG ages and clade assignments. Supplementary Table 7. Half-life data for *A. thaliana* RBPs. Supplementary Table 8. Deadenylation assay quantification.

Supplementary Code 1 Extended profile HMM for the RRM domain.

## Data Availability

RNAcompete raw and normalized intensity data are available on Gene Expression Omnibus (http://www.ncbi.nlm.nih.gov/geo/) under accession number GSE192895 (ref. ^67^). The browsable database of RBP motifs is available at https://cisbp.org/rna. Raw microarray data, array design information, 7-mer *z* scores, RNAcompete quality control plots, RBP motifs, *z-*score bootstrap analysis results and JPLE training data are available at https://hugheslab.ccbr.utoronto.ca/supplementary-data/RBPZoo/.

## Code availability

The code written to perform the analysis in this paper is available on GitHub at https://github.com/LXsasse/RBPbinding (ref. ^68^).
