# Supplementary material for: A resource of RNA-binding protein motifs across eukaryotes reveals evolutionary dynamics and gene-regulatory function
Source: Nat Biotechnol. 2025 Jul 25;44(7):1226–36. doi: 10.1038/s41587-025-02733-6 (PMC12990809; doi:10.1038/s41587-025-02733-6)
Supplement: Supplementary file 1 — Supplementary Notes 1–7. [file 41587_2025_2733_MOESM1_ESM.pdf]

nature biotechnology

Supplementary information

https://doi.org/10.1038/s41587-025-02733-6

A resource of RNA-binding protein motifs across eukaryotes reveals evolutionary dynamics and gene-regulatory function

In the format provided by the authors and unedited

Table of Contents

Supplementary Note 1: Generating an extended RRM profile HMM 2

Supplementary Note 2: Selecting RBPs to assay with RNAcompete 3

Supplementary Note 3: RNAcompete experimental protocol........ 4

Supplementary Note 4: Affinity Regression 7

Supplementary Note 5: Baseline models for interface characterization........ 8

Supplementary Note 6: Refinement of initial CRMG set........ 9

Supplementary Note 7: NOT6:NOT7 deadenylase heterodimer purification 11

References 12

Supplementary Note 1

Generating an extended RRM profile HMM

We extracted all available X-ray and NMR structures of RRMs in complex with RNA from PDB[1] and identified contacts between residues and nucleic acids using COCOMAPS[2]. From these results, we observed that the Pfam RRM_1 pHMM does not include all residues that contact RNA. To generate a new pHMM for the RRM domain, we used the full amino acid sequences of the PDB RRM structures to generate a structure-based (i.e., 3D) multiple sequence alignment (MSA) using the EXPRESSO/3D-COFFEE mode of T-COFFEE with SAP as the structural aligner[3,4]. To avoid sequence bias, we trimmed the final 3D MSA so that no pair of RRM sequences had an AA SID > 80%. We redefined the limits of the RRM domain to include residues that are in contact with RNA in at least one RRM structure while respecting the secondary structure elements representing the known fold of RRM domains. We used the trimmed 3D MSA to generate a pHMM using HMMER[5], resulting in an extended version of the standard Pfam RRM_1 pHMM that captures 15 additional residues (**Supplementary Code 1**).

Supplementary Note 2

Selecting RBPs to assay with RNAcompete

We employed four different strategies to select RBPs to assay with RNAcompete:

(1) To provide sufficient JPLE training data covering various AA similarity ranges, we selected RBPs with differing levels of AA SID to previously characterized RBPs[6]. To this end, we iteratively selected RBPs from the 45 species to evenly populate nine bins of pairwise AA SID (10-19.99% up to 90-99.99%), for each of the RRM and KH RBP families, such that we had at least ten comparisons per RBP family, per bin.

(2) We next sought to balance numbers across the major eukaryotic clades. For each major clade (metazoans, plants, fungi, and other eukaryotes), we selected the single RBP for which the largest number of other RBPs are >70% identical in their RBR. This level was chosen based on the previously established threshold for AA SID-based motif inference[6].

(3) To improve sampling of RBPs from diverse model organisms, which we reasoned would be useful to the largest number of investigators, we obtained data for 32 *Cannabis sativa* (plant), 18 *Caenorhabditis elegans* (nematode), 17 *Leishmania major* (parasitic protist), 14 *Homo sapiens* (primate), and 10 *Tetraodon nigroviridis* (fish) RBPs, selected to represent a diversity of RBPs from these species.

(4) To generally increase the total number of inferred motifs, we also selected additional uncharacterized RBPs with RBRs that are >70% identical to the largest number of other RBPs, regardless of eukaryotic clade.

Supplementary Note 3

RNAcompete experimental protocol

Synthesis of DNA and RNA Pool. RNA was synthesized from dsDNA pool templates generated from an Agilent 244K microarray (AMADID #024519). To generate the dsDNA pool, 8 nmoles of denatured, snap-cooled T7 promoter 5′ end labeled with Cy3 (5′- Cy3-CTAATACGACTCACTATTAG; Integrated DNA Technologies (IDT)) was annealed to an Agilent 244K microarray in hybridization buffer (10 mM Tris-HCl pH 7.5, 1 M NaCl, 0.5% Triton X-100, 0.75 mM DTT) for 4 hours at 30 °C and then washed — once with Wash Buffer 1 (6x SSPE/0.05% Triton X-100) and once with Wash Buffer 2 (0.06x SSPE) — to remove unbound primer. Arrays were scanned (Axon Genepix 4000B) for Cy3 fluorescence to confirm primer hybridization. Next, ssDNA microarray probes were made double stranded by enzymatic primer extension using 50 Units of Klenow Fragment (3'-5' exo-) (New England Biolabs (NEB), M0212S), 36 Units T4 DNA Polymerase (NEB, M0203S), and 1x BSA (NEB) in NEB Buffer #2 (10 mM Tris-HCl pH 7.9, 50 mM NaCl, 10 mM $MgCl_2$, 1 mM DTT, 100 μM dNTPs) at 30 °C for 30 min. The microarray slide was washed twice in Wash Buffer 3 (10 mM Tris-Cl pH 7.5, 50 mM NaCl, 10 mM $MgCl_2$) and dsDNA microarray probes were ligated to 15 nmoles of dsDNA linker using 18,000 Units of T4 DNA Ligase (NEB, M0202T) overnight at 16 °C. The dsDNA linker was generated by annealing a 5′-phosphate and dideoxycytosine (ddC) terminated oligo to a 5′-Cy5 labeled oligo (5′-P-T<u>GAAGAGC</u>GAGCGGATACAG-ddC, 5′-Cy5-CTGTATCCGCTC<u>GCTCTTC</u>A; core BspQI site is underlined, IDT). The microarray slide was washed twice in Wash Buffer 3 and linker ligation to the dsDNA microarray probes was verified by scanning for Cy5 fluorescence (Axon Genepix 4000B). ssDNA was stripped (twice) from the microarray by incubating with 20 mM NaOH at 65 °C for 20 minutes. The array was scanned for Cy3 and Cy5 fluorescence to visualize microarray stripping efficiency. Stripped ssDNA samples were pooled, precipitated with ethanol and PCR amplified using common primers (5′-CTAATACGACTCACTATTAG, 5′-CTGTATCCGCTCGCTCTTCA; IDT). The amplified dsDNA pool was isolated from agarose gels using a Qiaex II Gel Extraction Kit (Qiagen) and digested using 20 Units of BspQI (NEB, R3712S) to remove the linker. To synthesize RNA, purified BspQI-digested dsDNA pool (gel extracted using a Qiaex II Gel Extraction Kit,

followed by phenol-chloroform extraction and ethanol precipitation) was used as template in a T7 MegaScript transcription reaction (ThermoFisher, AM1334). Transcription reactions were extensively digested with DNase I to remove template DNA and the RNA pool was purified using a Sephadex G-25 spin column (GE Healthcare), phenol-chloroform extracted, precipitated with ethanol, and quantified spectrophotometrically (Nanodrop).

GST-tagged RBP Purification. GST-tagged RBP expression constructs were transformed into *Escherichia coli* C41 cells (Lucigen) and protein expression was induced by adding IPTG (1mM final) to log phase cell culture and incubating overnight at 16 °C. Cells were collected and resuspended in Lysis Buffer (20 mM HEPES pH 7.5, 0.1 mM EDTA, 1 M NaCl, 10 mM β-mercaptoethanol, 1 mM PMSF, 0.5% Triton X-100), and lysed with 15 mg of lysozyme (BioShop) and sonicated (MisoNix 3000 Sonicator (Mandel)). Lysed cell extracts were added to High Performance Glutathione Sepharose beads (GE Healthcare) and incubated for 2 hours at 4°C. GST beads were washed once in Buffer D (0.5 M NaCl, 10 mM β-mercaptoethanol) and twice in Buffer C (20 mM HEPES pH 7.5, 0.1 M NaCl, 0.1 mM EDTA, 10 mM β-mercaptoethanol). GST-tagged RBPs were eluted for 2 hours at 4 °C with Elution Buffer (50 mM Tris-HCl pH 8.8, 250 mM NaCl, 30 mM reduced glutathione, 10 mM β-mercaptoethanol, and 20% glycerol). Protein concentration and purity were estimated by SDS-PAGE and Bradford assay.

RNAcompete Assay. The RNA pool used in RNAcompete experiments contains 241,399 non-random RNA sequences, 30-41 nucleotides in length. RNA probes were designed to possess low probabilities for base pairing (i.e., the vast majority are single-stranded) and to represent each 7-mer at least 310 times. During RNAcompete experiments, the RNA pool was heated to 90 °C for 2 min, 65°C for 10 minutes, 37 °C for 10 minutes, and then kept at room temperature. Purified GST-tagged RBPs (20 nM) and RNA (1.5 μM) were incubated in Binding Buffer (1 mL final volume; 20 mM HEPES pH 7.8, 70 mM KCl, 10 mM NaCl, 10% glycerol, 2 mM DTT, 0.1 μg/μL Ultrapure BSA (ThermoFisher, AM2616)) containing 20 μL Glutathione Sepharose 4B (GE Healthcare) beads (pre-washed 3 times in binding buffer) for 30 min at 4 °C. GST beads were washed four times for 2 min with Binding Buffer to remove unbound RNAs. Bound RNAs were eluted/purified by adding 200 μL of STE (1% SDS, 10 mM Tris-Cl, 2

mM EDTA), heating to 95 °C for 2 min, extracting with phenol-chloroform and precipitating with ethanol in the presence of 20 μg of glycogen (ThermoFisher, R0551).

Microarray Hybridization. To measure the abundance of RNA bound by individual RBPs, RNA pulldown samples were directly labeled with Cy3 or Cy5 with a Cy3/Cy5 Labeling Kit (Kreatech, EA-021) using the manufacturer recommendations. Labeled RNA were precipitated with ethanol, resuspended in 8 μl of RNase-free water, combined (one Cy3- and one Cy5-labeled pulldown RNA from 2 different RNAcompete experiments per microarray), added to 103 μl of Hybridization Buffer (1 M NaCl, 0.5% sodium N-lauroyl sarcosine (SLS), 50 mM methyl ethane sulfonate, pH 6.5, 50% formamide), heated at 65 °C for 5 min, added to 40 μg of denatured salmon sperm DNA (Sigma), and loaded into a microarray hybridization chamber. Hybridizations on Agilent 244K microarrays (AMADID #024519) were carried out for 20 hours at 42 °C and washed for 30 seconds in 6 × SSPE, 0.005% SLS, and 30 seconds in 0.06 × SSPE in a HS4800 Pro Hybridization Station (Tecan). Microarrays were scanned with an Axon Genepix 4000B microarray scanner at 5 μm resolution and saved as TIFF images. Imagene software was used to capture the fluorescence of Cy3- or Cy5-labeled RNA hybridized to Agilent 244K microarrays.

Supplementary Note 4

Affinity Regression

Affinity regression (AR) is a machine-learning approach designed for predicting RNA specificities of RBPs[7]. Instead of modeling the direct mapping between ***P*** and ***R***, however, AR learns the interaction, ***A***, between RBP amino acid 4-mer counts ***P*** and RNA 5-mer counts ***D*** to reconstruct ***R*** during training:

$$\boldsymbol{DAP^T} \approx \boldsymbol{R^T}$$

, where $\boldsymbol{D} \in \mathbb{R}^{r \times r'}$, $\boldsymbol{A} \in \mathbb{R}^{r' \times p}$, $\boldsymbol{P} \in \mathbb{R}^{n \times p}$, $\boldsymbol{R} \in \mathbb{R}^{n \times r}$. We experimented with different formulations of ***D, A,*** and ***P*** and opted for the best performer, where *r* is the number of RNAcompete RNA 7-mers, *r'* is the number of all unique sub-[1-5]-mers within the RNA 7-mers, *p* is the number of RBP amino acid 4-mers, and *n* is the number of training set RBPs. The binary matrix ***D*** indicates whether a given sub-[1-5]-mer is present in each RNA 7-mer. Instead of optimizing ***A*** on the above equation, both sides are multiplied by ***R***, enabling AR to learn the similarity between the RNA specificities of RBPs, i.e.,

$$\boldsymbol{RDAP^T} \approx \boldsymbol{RR^T}$$

Like JPLE, AR employs SVD to denoise data and reduce computational costs. ***RD*** and ***P*** undergo SVD, retaining singular values and vectors contributing to at least 90% and 95% of their respective variances:

$$\boldsymbol{RD} \approx \boldsymbol{U_{RD}'\Sigma_{RD}'V_{RD}'^T}$$

$$\boldsymbol{P} \approx \boldsymbol{U_P'\Sigma_P'V_P'^T}$$

Finally, ***A*** is solved with L_2 regularization:

$$\text{argmin}_{\boldsymbol{A_{RD,P}}} \,||\, \text{vec}(\boldsymbol{RR^T}) - (\boldsymbol{V_P'} \otimes \boldsymbol{U_{RD}'})\, \text{vec}(\boldsymbol{A_{RD,P}})\, ||_2^2 + \lambda \,||\, \text{vec}(\boldsymbol{A_{RD,P}})||_2$$

, where $\boldsymbol{A_{RD,P}} = \boldsymbol{\Sigma_{RD}'V_{RD}'^T A}(\boldsymbol{U_P'\Sigma_P'})^{\mathrm{T}}$ and $\lambda = 1 / n^2$. To reconstruct the RNA-binding profile $\boldsymbol{r_u}^*$ of an uncharacterized RBP, the "mapping reconstruction" approach, similar to local decoding in a JPLE protein query, is used by taking the average of the ***r****'s* of all training set RBPs, weighted by the similarity between the training set and uncharacterized RBPs.

Supplementary Note 5

Baseline models for interface characterization

To compute protein sequence conservation, we performed MSA between the RBR sequence of each PDB entry and all RBR sequences from 690 species in CisBP-RNA, and retained those with greater than 30% AA SID to the PDB sequence. We then computed the Jensen-Shannon divergence for each residue in the MSA as a measure for per-residue conservation[8].

For the random forest model, we combined several distinct sequence features: conservation as described above, a position-specific scoring matrix (PSSM) derived from the same protein sequence alignment, physicochemical features (retrieved from [9]), and one-hot amino acid identity[10-12]. The random forest model performed interface predictions for the residue in the centre of a window of five residues. The features of these five residues were summed and provided to the model. The model was trained with LOOCV, focusing only on structures that shared less than 30% AA SID with the tested PDB entry to avoid recalling interface residues from homologous sequences.

To compare the three methods for interface characterization, we computed the Area Under the Receiver Operating Characteristic (AUROC) and the Area Under the Precision-Recall (AUPR) curve over all residues, with residues ranked by their RIS, conservation score, or random forest prediction value.

Supplementary Note 6

Refinement of initial CRMG set

Examining the initial draft of the CRMGs, we found that some of the initial clusters consisted of two or more groups of highly interconnected RBRs, with the groups connected by a single "transitioning" RBP or one, or a small number of links. We reasoned that these inter-group links were likely false-positives, potentially due to fusions of RBDs from distinct RBPs, and further split the connected component using a procedure that ensured that the resulting CRMGs both were consistent with the phylogeny and had an average e-dist < 0.2 between all pairs of RBRs. To do so, we applied species-tree guided agglomerative clustering (average linkage) on JPLE e-dist, separately to each initial CRMG.

Starting from the two most closely related species in the CRMG, we move the species tree upwards (i.e., further back in time) to the last common ancestor of the CRMG and at each branch point we check if the average e-dist between all descendent RBRs is below 0.2. If at any branch point, the average e-dist is > 0.2, the CRMG is split into two CRMGs. Additionally, we wanted to avoid retention of a CRMG with the highly unlikely evolutionary event that it was lost independently in two or more subsequent branch points. To do so we split a CRMG if a branch point and a subsequent descendent branch each contain a descendant branch that does not contain any RBPs in the CRMG (i.e., it was independently lost twice). This step separated the 2,463 initial clusters into 3,095 *post-split* CRMGs whose e-dists to one another were all > 0.2.

We then merged some post-split clusters in order to combine paralogs within a species that may share RNA-sequence specificity but were not part of the same connected component in the highest homology network (which only connects RBRs between different species). To identify these paralogous groups, we computed the average JPLE distance between all pairs of post-split CRMGs containing RBRs in the same species and merge any post-split CRMGs with average e-dist distance < 0.2. This step reduces the 3,095 post-split CRMGs to 2,866 paralog-merged CRMGs.

Although JPLE is able to correctly distinguish binding similarities between most RBRs, we were able to identify cases of false positives and negative groupings in the paralog-merged

CRMGs based on other features. First, single RBRs are removed from a paralog-merged CRMG if removing the RBR changed the common ancestor of the CRMG and if the RBR did not possess a one-to-one triangle orthology connection to any other pair of RBRs in the CRMG. This step generated 14 additional, single RBR groups, thus affecting only 0.5% of clusters. To account for potential false negatives from potentially inaccurate JPLE distances in low-confidence regions (i.e., far from training set RBPs), we combined CRMGs if on average they shared more than 70% AA SID. This step caused the merging of 417 (14.5%) mainly single RBR CRMGs into another CRMG. After this step, we were left with the final version of the CRMGs.

Supplementary Note 7

NOT6:NOT7 deadenylase heterodimer purification

NOT6:NOT7 heterodimer was co-expressed in *E. coli* BL21(DE3) Star cells (Thermo Fisher Scientific) in LB medium at 20 °C. NOT6 carried N-terminal His_6-tag and NOT7 carried His_6-SUMO-tag. Cells were lysed by sonication in a buffer containing 50 mM potassium phosphate pH 7.5, 300 mM NaCl and 25 mM imidazole. The cleared lysate was loaded onto a Ni-charged HiTrap IMAC column (Cytiva) and eluted from the column by using the same buffer supplemented with 250 mM imidazole. Tags were then cleaved off overnight by TEV protease. Then, the NOT6:NOT7 heterodimer was eluted on a Superdex 200 26/600 gel filtration column (Cytiva) equilibrated in a buffer containing 10 mM HEPES/NaOH pH 7.5, 200 mM NaCl and 2 mM DTT. For the ion exchange chromatography step, the NaCl concentration was diluted to 100 mM, and the complex was loaded onto a MonoQ 10/100 GL column (Cytiva). Subsequently, the complex was eluted by a linear gradient to buffer containing 10 mM HEPES/NaOH pH 7.5, 1 M NaCl and 2 mM DTT. The purified NOT6:NOT7 complex was concentrated to ~4 mg/ml, flash-frozen and stored at −80 °C.

References

1. Berman, H.M. *et al.* The Protein Data Bank. *Nucleic Acids Res* **28**, 235-42 (2000).
2. Vangone, A., Spinelli, R., Scarano, V., Cavallo, L. & Oliva, R. COCOMAPS: a web application to analyze and visualize contacts at the interface of biomolecular complexes. *Bioinformatics* **27**, 2915-6 (2011).
3. Poirot, O., Suhre, K., Abergel, C., O'Toole, E. & Notredame, C. 3DCoffee@igs: a web server for combining sequences and structures into a multiple sequence alignment. *Nucleic Acids Res* **32**, W37-40 (2004).
4. Armougom, F. *et al.* Expresso: automatic incorporation of structural information in multiple sequence alignments using 3D-Coffee. *Nucleic Acids Res* **34**, W604-8 (2006).
5. Eddy, S.R. A new generation of homology search tools based on probabilistic inference. *Genome Inform* **23**, 205-11 (2009).
6. Ray, D. *et al.* A compendium of RNA-binding motifs for decoding gene regulation. *Nature* **499**, 172-7 (2013).
7. Pelossof, R. *et al.* Affinity regression predicts the recognition code of nucleic acid-binding proteins. *Nat Biotechnol* **33**, 1242-1249 (2015).
8. Capra, J.A. & Singh, M. Predicting functionally important residues from sequence conservation. *Bioinformatics* **23**, 1875-82 (2007).
9. Li, Z., Tang, J. & Guo, F. Identification of 14-3-3 Proteins Phosphopeptide-Binding Specificity Using an Affinity-Based Computational Approach. *PLoS One* **11**, e0147467 (2016).
10. Kumar, M., Gromiha, M.M. & Raghava, G.P. Prediction of RNA binding sites in a protein using SVM and PSSM profile. *Proteins* **71**, 189-94 (2008).
11. Zhang, X. & Liu, S. RBPPred: predicting RNA-binding proteins from sequence using SVM. *Bioinformatics* **33**, 854-862 (2017).
12. Walia, R.R., El-Manzalawy, Y., Honavar, V.G. & Dobbs, D. Sequence-Based Prediction of RNA-Binding Residues in Proteins. *Methods Mol Biol* **1484**, 205-235 (2017).
