# Supplementary material for: A resource of RNA-binding protein motifs across eukaryotes reveals evolutionary dynamics and gene-regulatory function
Source: Nat Biotechnol. 2025 Jul 25;44(7):1226–36. doi: 10.1038/s41587-025-02733-6 (PMC12990809; doi:10.1038/s41587-025-02733-6)
Supplement: Supplementary file 2 — Reporting Summary [file 41587_2025_2733_MOESM2_ESM.pdf]

nature portfolio

Corresponding author(s): Quaid Morris
Timothy R. Hughes
Matthew T. Weirauch

Last updated by author(s): Jun 2, 2025

Reporting Summary

Nature Portfolio wishes to improve the reproducibility of the work that we publish. This form provides structure for consistency and transparency in reporting. For further information on Nature Portfolio policies, see our Editorial Policies and the Editorial Policy Checklist.

Statistics

For all statistical analyses, confirm that the following items are present in the figure legend, table legend, main text, or Methods section.

n/a	Confirmed	
☐	☒	The exact sample size (*n*) for each experimental group/condition, given as a discrete number and unit of measurement
☐	☒	A statement on whether measurements were taken from distinct samples or whether the same sample was measured repeatedly
☐	☒	The statistical test(s) used AND whether they are one- or two-sided<br>*Only common tests should be described solely by name; describe more complex techniques in the Methods section.*
☒	☐	A description of all covariates tested
☐	☒	A description of any assumptions or corrections, such as tests of normality and adjustment for multiple comparisons
☒	☐	A full description of the statistical parameters including central tendency (e.g. means) or other basic estimates (e.g. regression coefficient) AND variation (e.g. standard deviation) or associated estimates of uncertainty (e.g. confidence intervals)
☐	☒	For null hypothesis testing, the test statistic (e.g. *F*, *t*, *r*) with confidence intervals, effect sizes, degrees of freedom and *P* value noted<br>*Give P values as exact values whenever suitable.*
☒	☐	For Bayesian analysis, information on the choice of priors and Markov chain Monte Carlo settings
☐	☒	For hierarchical and complex designs, identification of the appropriate level for tests and full reporting of outcomes
☐	☒	Estimates of effect sizes (e.g. Cohen's *d*, Pearson's *r*), indicating how they were calculated

Our web collection on statistics for biologists contains articles on many of the points above.

Software and code

Policy information about availability of computer code

Data collection	Detection of microarray Cy3 and Cy5 intensities were measured using ImaGene 8.0 (BioDiscovery).<br>Detection of fluorescence of RNA gels: Sapphire FL v1.3.2.1027 (Azure Biosystems)
Data analysis	The code written to perform the analysis in this paper is available on GitHub: https://github.com/LXsasse/RBPbinding<br><br>Additional code and software used for computational analysis.<br>HMMER3 (3.1b1), COCOMAPS (PMID: 21873642), T-Coffee (PMIDs: 15215345, 16845081), RF2NA (v0.2), Alpha Fold (v3.0.0), score_conservation.py (PMID: 17519246), clustalOmega (v1.2.4), SCRATCH (SCRATCH-1D release 1.2), ETE Toolkit (ete3), RNAfold webserver (http://rna.tbi.univie.ac.at/cgi-bin/RNAWebSuite/RNAfold.cgi, accessed Dec 2024), QGRS Mapper webserver (https://bioinformatics.ramapo.edu/QGRS/index.php, accessed Dec 2024).<br><br>Data analysis of deadenylation assays was conducted with AzureSpot Pro 1.4-583 (Azure Biosystems) and GraphPad Prism v10.3.1.

For manuscripts utilizing custom algorithms or software that are central to the research but not yet described in published literature, software must be made available to editors and reviewers. We strongly encourage code deposition in a community repository (e.g. GitHub). See the Nature Portfolio guidelines for submitting code & software for further information.

Data

Policy information about availability of data

All manuscripts must include a data availability statement. This statement should provide the following information, where applicable:

- Accession codes, unique identifiers, or web links for publicly available datasets
- A description of any restrictions on data availability
- For clinical datasets or third party data, please ensure that the statement adheres to our policy

RNAcompete raw and normalized intensity data is available at GEO (http://www.ncbi.nlm.nih.gov/geo/) under accession number GSE192895. The browsable database of RBP motifs is available at https://cisbp.org/rna. Raw microarray data, array design information, 7-mer Z-scores, RNAcompete QC plots, RBP motifs, Z-score bootstrap analysis results, and JPLE training data are available at https://hugheslab.ccbr.utoronto.ca/supplementary-data/RBPZoo/.

The following publicly available data was retrieved for use in our analyses:
Publicly available data used for analysis:
Pfam pHMMs: CSD (PF00313.18), KH_1 (PF00013.25), La (PF05383.13), NHL (PF01436.17), PUF (PF00806.15), S1 (PF00575.19), SAM_1 (PF00536.26), YTH (PF04146.11), zf-CCCH (PF00642.20), zf-CCHC (PF00098.19), zf-CCHH (PF10283.5), and zf-RanBP (PF00641.14).
PDB structures: 1cvj:A, 2adb:A, 2adc:A, 2kfy:A, 2kg0:A, 2kg1:A, 2km8:C, 2leb:A, 2m8d:B, 2mgz:A, 2mgz:B, 2mkk:A, 2mqo:A, 2mqp:A, 2mqq:A, 2mxy:A, 2n82:B, 2rqc:A, 2rs2:A, 4bs2:A, 4c4w:E, 4ed5:B, 4lmz:A, 4qqb:A, 5en1:A, 5x3z:A
A. thaliana half-life data: Supplemental Table 5 (PMID: 18024567).
Evolutionary distances between species: TimeTree (version 4).
Pretrained TAPE models: UniRep (https://s3.amazonaws.com/songlabdata/proteindata/pytorch-models/unirep-base-config.json, https://s3.amazonaws.com/songlabdata/proteindata/pytorch-models/unirep-base-pytorch_model.bin) Transformer (i.e., bert) (https://s3.amazonaws.com/songlabdata/proteindata/pytorch-models/bert-base-config.json, https://s3.amazonaws.com/songlabdata/proteindata/pytorch-models/bert-base-pytorch_model.bin).
Physicochemical features: Table 2 (PMID: 26828594).

Research involving human participants, their data, or biological material

Policy information about studies with human participants or human data. See also policy information about sex, gender (identity/presentation), and sexual orientation and race, ethnicity and racism.

Reporting on sex and gender	N/A
Reporting on race, ethnicity, or other socially relevant groupings	N/A
Population characteristics	N/A
Recruitment	N/A
Ethics oversight	N/A

Note that full information on the approval of the study protocol must also be provided in the manuscript.

Field-specific reporting

Please select the one below that is the best fit for your research. If you are not sure, read the appropriate sections before making your selection.

☒ Life sciences ☐ Behavioural & social sciences ☐ Ecological, evolutionary & environmental sciences

For a reference copy of the document with all sections, see nature.com/documents/nr-reporting-summary-flat.pdf

Life sciences study design

All studies must disclose on these points even when the disclosure is negative.

Sample size	We used a semi-automated pipeline (using specific criteria such as RNA-binding domain type, amino acid sequence identity, presence in model organisms and/or underrepresented eukaryotic clades, etc.) to identify a panel of 277 RBPs across 45 species . This endeavour represents the 2nd largest study on RNA-binding to date which underscores its broad scope.
Data exclusions	174 RNAcompete experiments were deemed successful based on previously established success criteria (PMID: 27956239). The remaining 103 experiments were excluded from further analysis.
Replication	RNAcompete experiments have built in replication. Each microarray is composed of two sets of probes, each of which contains at least 155 copies of all RNA 5-mers. We performed automated and expert curation of the microarray data -- these QC steps ensure that the replicate sets were consistent and yielded highly similar results. Experiments that did not meet our stringent QC were deemed to be unsuccessful and were excluded from further analysis.<br>The deadenylation assay was performed in triplicate. All replicates were successful.

Randomization	The experimental assays were performed independently and do not influence each other. Individual RBPs are assayed in the order that they were purified — RBPs were not randomized but they were also not grouped in any particular way.
Blinding	Blinding is not relevant for this study. We analyzed a single group of 277 individual RNA-binding proteins using RNAcompete -- an in vitro assay. Identical experimental and data analysis pipelines are used for all RBPs regardless of whether their identities are known or unknown.

Reporting for specific materials, systems and methods

We require information from authors about some types of materials, experimental systems and methods used in many studies. Here, indicate whether each material, system or method listed is relevant to your study. If you are not sure if a list item applies to your research, read the appropriate section before selecting a response.

Materials & experimental systems

n/a	Involved in the study
☒	☐ Antibodies
☒	☐ Eukaryotic cell lines
☒	☐ Palaeontology and archaeology
☒	☐ Animals and other organisms
☒	☐ Clinical data
☒	☐ Dual use research of concern
☒	☐ Plants

Methods

n/a	Involved in the study
☒	☐ ChIP-seq
☒	☐ Flow cytometry
☒	☐ MRI-based neuroimaging

Plants

Seed stocks	*Report on the source of all seed stocks or other plant material used. If applicable, state the seed stock centre and catalogue number. If plant specimens were collected from the field, describe the collection location, date and sampling procedures.*
Novel plant genotypes	*Describe the methods by which all novel plant genotypes were produced. This includes those generated by transgenic approaches, gene editing, chemical/radiation-based mutagenesis and hybridization. For transgenic lines, describe the transformation method, the number of independent lines analyzed and the generation upon which experiments were performed. For gene-edited lines, describe the editor used, the endogenous sequence targeted for editing, the targeting guide RNA sequence (if applicable) and how the editor was applied.*
Authentication	*Describe any authentication procedures for each seed stock used or novel genotype generated. Describe any experiments used to assess the effect of a mutation and, where applicable, how potential secondary effects (e.g. second site T-DNA insertions, mosiacism, off-target gene editing) were examined.*
